# Periviscerokinin (*Cap*_*2b*_; *CAPA)* receptor silencing in females of *Rhipicephalus microplus* reduces survival, weight and reproductive output

**DOI:** 10.1186/s13071-022-05457-7

**Published:** 2022-10-06

**Authors:** Juan P. Wulff, Kevin B. Temeyer, Jason P. Tidwell, Kristie G. Schlechte, Kimberly H. Lohmeyer, Patricia V. Pietrantonio

**Affiliations:** 1 Department of Entomology, Texas A&M University, College Station, TX 77843-2475 USA; 2 Knipling-Bushland U.S. Livestock Insects Research Laboratory and Veterinary Pest Genomics Center, United States Department of Agriculture–Agricultural Research Service, 2700 Fredericksburg Road, Kerrville, TX 78028-9184 USA; 3 Cattle Fever Tick Research Laboratory, United States Department of Agriculture–Agricultural Research Service, 22675 N. Moorefield Rd. Building 6419, Edinburg, TX 78541-5033 USA

**Keywords:** Cardioacceleratory peptide 2b (CAP_2b_)/periviscerokinins (PVK) neuropeptide family, *CAPA* gene, RNA interference, Cattle fever tick, Southern cattle tick, Asian blue tick, Tick survival, Tick reproduction, Egg hatching, Tick feeding

## Abstract

**Background:**

The cattle fever tick, *Rhipicephalus* (*Boophilus*) *microplus*, is a vector of pathogens causative of babesiosis and anaplasmosis, both highly lethal bovine diseases that affect cattle worldwide. In Ecdysozoa, neuropeptides and their G-protein-coupled receptors play a critical integrative role in the regulation of all physiological processes. However, the physiological activity of many neuropeptides is still unknown in ticks. Periviscerokinins (CAP_2b_/PVKs) are neuropeptides associated with myotropic and diuretic activities in insects. These peptides have been identified only in a few tick species, such as *Ixodes ricinus*, *Ixodes scapularis* and *R. microplus,* and their cognate receptor only characterized for the last two.

**Methods:**

Expression of the periviscerokinin receptor (*Rhimi-CAP*_*2b*_*R*) was investigated throughout the developmental stages of *R. microplus* and silenced by RNA interference (RNAi) in the females. In a first experiment, three double-stranded (ds) RNAs, named ds680-805, ds956-1109 and ds1102-1200, respectively, were tested in vivo. All three caused phenotypic effects, but only the last one was chosen for subsequent experiments. Resulting RNAi phenotypic variables were compared to those of negative controls, both non-injected and dsRNA beta-lactamase-injected ticks, and to positive controls injected with beta-actin dsRNA. *Rhimi-CAP*_*2b*_*R* silencing was verified by quantitative reverse-transcriptase PCR in whole females and dissected tissues.

**Results:**

*Rhimi-CAP*_*2b*_*R* transcript expression was detected throughout all developmental stages*. Rhimi-CAP*_*2b*_*R* silencing was associated with increased female mortality, decreased weight of surviving females and of egg masses, a delayed egg incubation period and decreased egg hatching (*P* < 0.05).

**Conclusions:**

CAP_2b_/PVKs appear to be associated with the regulation of female feeding, reproduction and survival. Since the *Rhimi-CAP*_*2b*_*R* loss of function was detrimental to females, the discovery of antagonistic molecules of the CAP_2b_/PVK signaling system should cause similar effects. Our results point to this signaling system as a promising target for tick control.

**Graphical Abstract:**

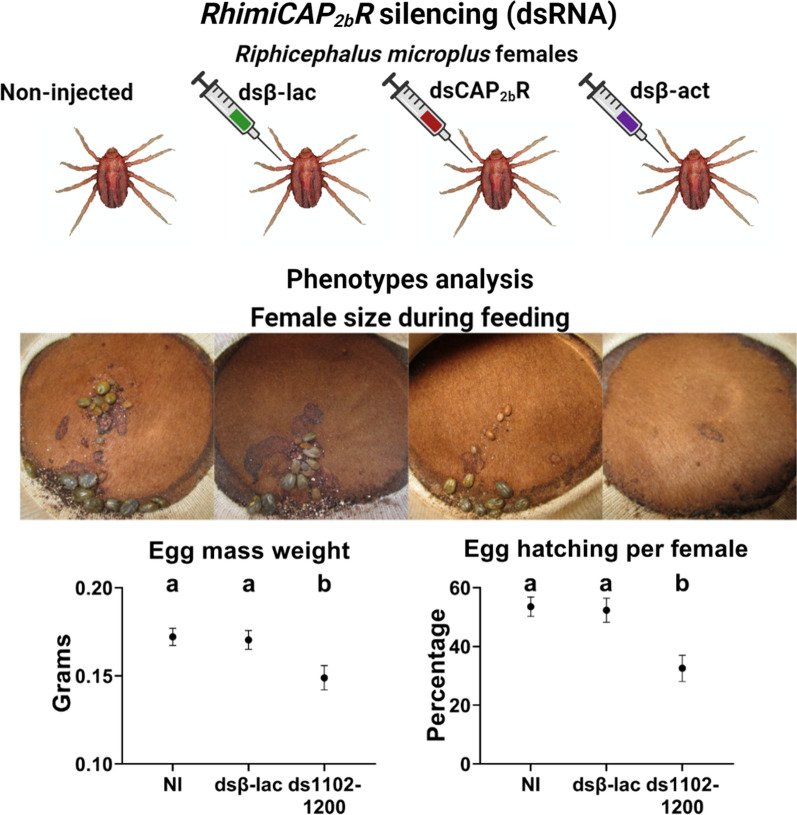

**Supplementary Information:**

The online version contains supplementary material available at 10.1186/s13071-022-05457-7.

## Background

Ticks (Acari: Ixodidae) are vectors of pathogens causing diseases in humans and livestock worldwide [1]. Tick infestation also causes monetary losses by direct stress effects on domestic and farm animal health [2, 3]. Further, the prevalence of tick-borne diseases has increased recently in humans and livestock, and tick distribution is continually expanding due to direct and indirect human actions, such as livestock transportation and climate change [3, 4].

The cattle fever tick (CFT) or Asian blue tick, *Rhipicephalus* (*Boophilu*s) *microplus*, known as the southern cattle tick in the USA, is the vector of pathogens causative of babesiosis and anaplasmosis, two highly lethal bovine diseases that affect cattle globally [5–7]. CFT control mainly focuses on pesticides that target the nervous system or that inhibit chitin deposition in the cuticle [8, 9]. However, the CFT populations worldwide have developed resistance to several pesticides, such as amidines (amitraz), organophosphates, pyrethroids, fluazuron and ivermectin [10–13]. The lack of recombinant vaccines that prevent deadly cattle babesiosis [14] reinforces the critical need to validate novel and selective pesticide targets to control this and other tick species.

In the Ecdysozoa, neuropeptides and their receptors play a critical integrative role regulating all physiological processes, such as feeding, excretion, molting, diapause and mating [15, 16]. Neuropeptides have a ubiquitous role in insects as well, and many of them display a pleiotropic function [17]. Therefore, these and their receptors [G protein-coupled receptors (GPCRs)], have been suggested as potential targets for pest control [18, 19]. Among other arthropods, neuropeptides and their receptors may similarly have integrative functions in ticks (Acari: Ixodidae) [20, 21]. However, little is known about their physiological functions in comparison to other groups, such as insects and crustaceans [17, 22], beyond a few neuropeptides and cognate GPCRs characterized in ticks, such as those described in [23–29].

The study of neuropeptides and their GPCRs in ticks has also been suggested as a strategy for discovering novel selective targets for tick control [20]. A few GPCRs for small neuropeptides have been investigated in *R. microplus* through RNA interference (RNAi), and the results suggested a role in feeding and reproduction [29, 30]. High-throughput screens have been used to discover small molecule antagonists of the tick kinin receptor with the goal of reproducing in vivo the effects observed after kinin receptor silencing by RNAi [29, 31]. The myotropic activity of pyrokinins in feeding-related tissues of two tick species, *Rhipicephalus sanguineus* and *Ixodes scapularis*, was recently demonstrated [32].

In the present study we focus on another receptor for a small neuropeptide, the periviscerokinin (CAP_2b_/PVK) receptor of the southern cattle tick [25], for which the cognate ligand in insects is coded by the Capability gene (*CAPA*). The Capability/Pyrokinin (*CAPA/PK*) is an ancient endocrine signaling system in Ecdysozoa, and putatively homologous of the Vertebrata Neuromedin U system [33]. In insects, the ancestral *CAPA*/*PK* gene was duplicated and differentiated into two genes [34]; however, active peptides encoded under each gene can differ among species. In *Drosophila melanogaster*, the *CAPA* gene codes for two CAP_2b_/PVKs, namely CAPA-PVK-1 and -2, both with a PRV-amide ending [35], and for a single trypto-pyrokinin, CAPA-PK-3, with a C-terminal WFGPRL-amide ending [35], later designated CAPA pyrokinin Diapause Hormone 1 (DH-1) [34, 36]. The *Drosophila* hugin gene encodes only pyrokinins (PKs) [37]. A similar arrangement exists in *Tribolium castaneum*, where the *CAPA* gene codes for PVKs and the trypto-PK [38]. Regarding the insect CAPA-related peptide receptors, these have been characterized in several insect species (see, for example, [39–41]). The insect CAP_2b_/PVK GPCR belongs to PRX-amide peptide receptor family [33].

Regarding CAP_2b_/PVK function, in several insect species these peptides stimulate fluid secretion from Malpighian tubules [42–44] and act as antidiuretic hormones [44, 45]. In *Aedes aegypti*, CAP_2b_/PVKs display either diuretic or antidiuretic activity in isolated Malpighian tubules, depending on their concentration [46]. However, antidiuretic activity appears to be the main role of CAP_2b_/PVKs in *Ae. aegypti*, having a potent inhibitory activity against diuretic hormones [47]. The myotropic activity of CAP_2b_/PVKs was first discovered in *Manduca sexta* and has also been observed in several tissues of Blaberidae and Blattidae cockroaches [48]. In roaches, CAP_2b_/PVKs were initially detected from the abdominal neurohemal organs, designated perisympathetic organs (PSOs) or perivisceral organs, from which the name periviscerokinins derives. CAP_2b_/PVKs are neurohormones released from PSOs into the hemolymph, and their hormonal actions on different target tissues may differ between insect species; they may also be neuromodulators [49].

CAP_2b_/PVKs have been identified in only a few tick species, such as *Ixodes ricinus*, *I. scapularis* and *R. microplus* [50–52]. In *R. microplus,* two CAP_2b_/PVKs were identified in a cloned cDNA of the CAP_2b_/PVK precursor, namely Rhimi-CAPA-PVK1 [52] and Rhimi-CAPA-PVK2, with sequences pQGLIPFPRVa and pQLVPVIRNa, respectively [32]. CAPA-PVK2, which features the unusual PRN-amide C-terminal ending, was also observed in *R. sanguineus* and *I. scapularis* [32, 52]. In insects, the PRN-amide is an uncommon ending and has only been identified in Blattodea (e.g.: genera *Deropeltis* and *Periplaneta*), as reported in the DINeR neuropeptide insect database that analyzed 201 species and their 539 PVK corresponding sequences [53]. The tick CAP_2b_/PVK receptor has been annotated in *I. scapularis* [51] and *R. microplus* [25], but the complementary DNA (cDNA) was cloned and functionally analyzed only for the second species [25]. In *R. microplus,* the CAP_2b_/PVK receptor transcript was detected in synganglion, salivary gland, Malpighian tubules and the ovary of partially fed female ticks [25]. Regarding the physiological role of CAP_2b_/PVKs in ticks, a myotropic activity was observed in the hindgut of *I. scapularis* [54]. The expression of the CAP_2b_/PVK transcript receptor in Malpighian tubules and ovaries [25] may suggest a role in diuresis and reproduction, but these have not been studied.

We previously performed CAP_2b_/PVK immunolocalization in the synganglion of *I. ricinus* with an anti-PVK2 from *Periplaneta americana* [50, 55] and have also demonstrated the functionality of the *R. microplus* CAP_2b_/PVK receptor [25] and identified the cDNA of *R. microplus* CAP_2b_/PVK precursor [52]. From this precursor we determined that the Rhimi-CAPA-PVK1 sequence p**QG**LIPFPRVa, which activates the receptor at the nanomolar level (half maximal effective concentration [EC_50_] = 64 nM) [25], differs from the sequence previously identified by MALDI-TOF, **PA**LIPFPRVa, in their two N-terminal residues (highlighted in bold) [50] but identical to the *I. scapularis* PVK [25, 52]. In the present work, we used RNAi to investigate the role of the CAP_2b_/PVK receptor in females of the cattle tick *R. microplus* (*Rhimi-CAP*_*2b*_*R*).

## Methods

### Tick rearing and animal care

Ticks were reared at the Cattle Fever Tick (CFT) Research Laboratory (US Department of Agriculture-Agricultural Research Service [USDA-ARS]; Mission, TX, USA) under a cooperative agreement with Texas A&M AgriLife Research (College Station, TX, USA). Cattle used for tick production or gene silencing experiments were CFT-naïve Hereford, Charolais or Angus, each weighing from 136 to 182 kg. Cattle were vaccinated, dewormed and acclimated for 2 weeks at the USDA-ARS Knipling-Bushland US Livestock Insects Research Laboratory (Kerrville, TX, USA) prior to shipment to the USDA-ARS Cattle Fever Tick Research Laboratory (CFTRL), a bio-secure research facility near Edinburg, Texas, USA. Cattle used for routine tick rearing or RNAi experiments at the CFTRL were maintained under approved animal use protocols (AUPs). All procedures for handling and treating animals were approved by the Texas A&M University (TAMU) Institutional Animal Care and Use Committee (IACUC) (TAMU AUP 2019-0197 EX under PVP, referring to IACUC USDA-ARS approved AUP 2021-12). Ticks used in this study were obtained from colonies of *R. microplus* acaricide-susceptible Deutch strain established from ticks collected in 2001 from an outbreak in Webb County, Texas, USA [56]. Filial generations F65, F68 and F80 were used for the RNAi experiments performed on January 2018, December 2019 and July 2021, respectively.

### RNA isolation, cDNA synthesis and quantitative reverse-transcriptase PCR analysis

Prior to RNA extraction, whole female ticks and tissues were disrupted using a Omni Bead Ruptor 12 homogenizer (Omni International, Inc., Waterbury, CT, USA) with equal proportions of 1.4- and 2.8-mm ceramic beads. Disruption of soft tissues, such as the female reproductive system, synganglion, Malpighian tubules and salivary glands, was for 1 min, and of the whole tick and carcass for 3 min, all at 5.65 Hz (m/s). Total RNA was extracted using the Zymo Quick-RNA™ Microprep Kit (Zymo Research, Irvine, CA, USA), according to the manufacturer's specifications. Two DNAse 1 (Deoxyribonuclease 1) steps were conducted: (i) during RNA extraction 30 U of DNAse 1 was added to the sample prior to 15 min of incubation at room temperature (RT); and (ii) after solubilization of the sample in nuclease-free water (NF-water) 5 U of DNAse 1 was added and incubation followed for 15 min at RT. After DNAse 1 treatment, the RNA Clean & Concentrator™-5 Kit (Zymo Research) was used to clean the sample (following the manufacturer's specifications), and the sample was recovered in 13 µl of NF-water. Clean total RNA (2 µl) was quantified spectrophotometrically using a Tecan Infinite M200 Pro plate reader (Tecan; Research Triangle Park, NC, USA).

Total RNA from whole ticks or tissues was used for cDNA synthesis. The reaction consisted of 150–200 ng of total RNA as template, 1 µl oligo-dT20 (50 µM) and 1 µl random hexamers (50 ng/µl) in 22 µl of final volume, and was performed using the SuperScript™ III First-Strand Synthesis System (Invitrogen, Thermo Fisher Scientific, Waltham, MA, USA) according to the manufacturer's specifications. The synthesized cDNA concentration was checked using 2 µl of a 1:10 diluted cDNA in a Tecan Infinite M200 Pro plate reader, and the undiluted cDNA was stored at − 20 °C until further use.

### Quantitative reverse-transcriptase PCR analyses for evaluation of expression and silencing

Quantitative reverse-transcriptase PCR (qRT-PCR) was performed in a reaction volume of 10 µl containing 5 µl PowerUp SYBR™ Green PCR Master Mix (Applied Biosystems, Thermo Fisher Scientific), 1 µl of a primer mix (300 nM final concentration of each primer), 2 µl of cDNA (40 ng/µl) and 2 µl of NF-water. All reactions were performed in duplicate. Real-time relative quantification was performed using the QuantStudio 6 Flex Real-Time PCR System (Applied Biosystems, Thermo Fisher Scientific). The conditions for the qRT-PCR cycles consisted of an initial denaturation step at 95 °C for 10 min, followed by 40 cycles at 95 °C for 15 s and 60 °C for 60 s. All oligonucleotide primers (Table 1) used for qRT-PCR were synthesized by Integrated DNA Technologies (IDT, Coralville, IA, USA).

**Table 1:** Oligonucleotide primers for cloning, double-stranded RNA synthesis and quantitative reverse-transcriptase PCR

Primer name	Oligo sequence 5′- 3′^a^	Notes
RmPerivis-200U19	ATCTGCTGTGCCGACACTG	*Rhimi-CAP*_*2b*_*R* PCR
RmPerivis-1716L21	GGACAGGGTATGGCGTTTATG	*Rhimi-CAP*_*2b*_*R* PCR
RmPerivis-388L18	CCGTATGGTGGCGTTGTC	5’-RACE-PCR
RmPerivis-195L20	GTCGGCACAGCAGATAGTCC	5’-RACE-PCR
Long-Univ.-RACE	CTAATACGACTCACTATAGGGCAAGCAGTGGTATCAACGCAGAGT	RACE-PCR
Short-Univ.-RACE	CTAATACGACTCACTATAGGGC	RACE-PCR
RmPeriviscR-680U20	CCCGCTTCAAGCTGCATGTG	*Rhimi-CAP*_*2b*_*R* dsRNA synthesis
RmPeriviscR-805L20	CCCGTCCACCATGCACTTTA	*Rhimi-CAP*_*2b*_*R* dsRNA synthesis
RmPeriviscR-680U20-T7	*AAAGGCCTTAATACGACTCACTATAGG*CCCGCTTCAAGCTGCATGTG	*Rhimi-CAP*_*2b*_*R* dsRNA synthesis
RmPeriviscR-805L20-T7	*AAAGGCCTTAATACGACTCACTATAGG*CCCGTCCACCATGCACTTTA	*Rhimi-CAP*_*2b*_*R* dsRNA synthesis
RmPeriviscR-956U20	GTGAAGGTTTCGCTTGAAGA	*Rhimi-CAP*_*2b*_*R* dsRNA synthesis
RmPeriviscR-1109L20	CCCTTCACAGTGTCGGCACA	*Rhimi-CAP*_*2b*_*R* dsRNA synthesis
RmPeriviscR-956U20-T7	*AAAGGCCTTAATACGACTCACTATAGG*TGAAGGTTTCGCTTGAAGA	*Rhimi-CAP*_*2b*_*R* dsRNA synthesis
RmPeriviscR-1109L20-T7	*AAAGGCCTTAATACGACTCACTATAGG*CCCTTCACAGTGTCGGCACA	*Rhimi-CAP*_*2b*_*R* dsRNA synthesis
RmPeriviscR-1102U20	TATCTGCTGTGCCGACACTG	*Rhimi-CAP*_*2b*_*R* dsRNA synthesis
RmPeriviscR-1200L20	CAGCACCGCAAGGATAATCA	*Rhimi-CAP*_*2b*_*R* dsRNA synthesis
RmPeriviscR-1102U20-T7	*AAAGGCCTTAATACGACTCACTATAGG*TATCTGCTGTGCCGACACTG	*Rhimi-CAP*_*2b*_*R* dsRNA synthesis
RmPeriviscR-1200L20-T7	*AAAGGCCTTAATACGACTCACTATAGG*CAGCACCGCAAGGATAATCA	*Rhimi-CAP*_*2b*_*R* dsRNA synthesis
Amp-fwd	CGCTGGTGAAAGTAAAATATG	beta-lactamase dsRNA synthesis [29]
Amp-rev	GCCGGGAAGCTAGAGTAAGTA	beta-lactamase dsRNA synthesis [29]
Amp-T7	*AAAGGCCTTAATACGACTCACTATAGG*CGCTGGTGAAAGTAAAATATG	beta-lactamase dsRNA synthesis [29]
Amp-T7	*AAAGGCCTTAATACGACTCACTATAGG*CCGGGAAGCTAGAGTAAGTA	beta-lactamase dsRNA synthesis [29]
BmBActin-1U20	TCCTCGTCCCTGGAGAAGTC	*Rhimi-ACTB* dsRNA synthesis [29]
BmBActin-285L18	GGGGGAGCGATGATCTTG	*Rhimi-ACTB* dsRNA synthesis [29]
BmbActin-1U20-T7	*AAAGGCCTTAATACGACTCACTATAGG*TCCTCGTCCCTGGAAGAAGTC	*Rhimi-ACTB* dsRNA synthesis [29]
BmbActin-285L18-T7	*AAAGGCCTTAATACGACTCACTATAGG*GGAGCGATGATCTTG	*Rhimi-ACTB* dsRNA synthesis [29]
RmCAP2b-qF1	ATGCGGGCCCTCATCAT	*Rhimi-CAP*_*2b*_*R* qRT-PCR [25]
RmCAP2b-qR1	AATGGCGGTTCCTGGTTAGG	*Rhimi-CAP*_*2b*_*R* qRT-PCR [25]
BmELF1a-88-F	CGTCTACAAGATTGGTGGCATT	*Rhimi-EF1A* qRT-PCR [57]
BmELF1a-196-R	CTCAGTGGTCAGGTTGGCAG	*Rhimi-EF1A* qRT-PCR [57]
RmRPS4-qF1	TCATCCTGCACCGCATCA	*Rhimi-RPS4* qRT-PCR [27]
RmRPS4-qR1	ACGCGGCACAGCTTGTACT	*Rhimi-RPS4* qRT-PCR [27]

*cDNA* Complementary DNA, *dsDNA* double-stranded DNA,* qRT-PCR* quantitative reverse-transcriptase PCR,* RACE *rapid amplification of cDNA ends,* Rhimi-CAP*_*2b*_*R* *Rhipicephalus* *microplus* periviscerokinin receptor

^a^Sequences in italics were added to the primers and are not part of the tick cDNA sequences

The reference genes selected for the qRT-PCR analysis had been previously tested on *R. microplus* on the same tissues and physiological conditions (3- to 5-day-old partially engorged females) [27, 29, 30]. These reference genes were elongation factor 1 alpha (*Rhimi-EF1A;* EW679365.1) and ribosomal protein S4 (*Rhimi-RPS4;* CV436347). The normalized relative quantity (NRQ) with respect to these reference genes was calculated for *Rhimi-CAP*_*2b*_*R* following the formulas in [58]. The NRQ values were subsequently utilized for estimation of fold change as ratios between the silenced ticks and controls (see section Tissue collection, RNA extraction, cDNA synthesis and qRT-PCR for evaluation of gene silencing.

### *Rhimi-CAP*_*2b*_*R* relative expression throughout different stages of development

*Rhimi-CAP*_*2b*_*R* relative expression throughout different stages of development was analyzed by qRT-PCR. The development stages were as follows: eggs, neolarvae, equal to first instar larva on the host [59], nymphs and adults. All ticks collected for the analyses were unfed, flash frozen within 24 h of emergence and kept in 500 µl of RNAlater™ (Invitrogen, Thermo Fisher Scientific) at − 80 °C until use. Nymphs and adults were obtained from two patches on a Hereford calf; each patch had been infested with approximately 250 mg of tick neolarvae. Engorged larvae were removed from the calf 6–7 days after infestation and allowed to molt in an environmental chamber at 25 ± 2 °C and a relative humidity (RH) of 95% [60]. Newly molted nymphs were placed on the animal and then removed as engorged nymphs 13–14 days post-infestation. Nymphs were placed in an environmental chamber maintained at the same temperature and RH as stated above until ecdysis. There were eight biological replicates (*n* = 8) per developmental stage and the replicates for each stage were as follows: (i) egg masses (approx. 100 mg [11.6 mg ± 0.9 mg] per replicate, mixed between 3 to 6 days after oviposition); (ii) neolarvae (10 whole bodies pooled per replicate); (iii) nymphs (5 whole bodies pooled per replicate); (iv) male (unmated, 2 whole bodies pooled per replicate); and (v) female (unmated, 1 whole body per replicate).

All ticks (F81) were kept under the same conditions as described in section Tick rearing and animal care. Total RNA extraction, cDNA synthesis, qRT-PCR conditions, reference genes and target gene (*Rhimi-CAP*_*2b*_*R*) were the same as those used for RNA isolation, cDNA synthesis and qRT-PCR analysis (see relevant sections), and for qRT-PCR analyses for evaluation of expression and silencing.

### *Rhimi-CAP*_*2b*_*R* gene silencing by RNAi

#### Synthesis of double-stranded RNAs for RNAi

To obtain cDNA as template to synthesize double-stranded RNA (dsRNA) for silencing experiments, the sequence of the cloned cDNA was first amplified by PCR, followed by amplification of the 5′-untranslated region (UTR) by PCR and cloning. These two cDNA amplifications were performed with different primers and using independently synthesized cDNAs. The steps followed are detailed below.

To amplify the receptor cloned cDNA, total RNA was extracted from *R. microplus* Deutch strain whole unfed females, using the ZR Tissue & Insect RNA Microprep™ Kit (Zymo Research), following the manufacturer's specifications. The extracted RNA was used for cDNA synthesis in a total reaction volume of 20 μl containing 1 µg of RNA as template and 1 µl oligo-dT20 (50 µM), using the SuperScript™ IV First-Strand Synthesis System (Invitrogen, Thermo Fisher Scientific) and following the manufacturer's specifications. Subsequently, *Rhimi-CAP*_*2b*_*R* cDNA sequence amplification was conducted by PCR, using the Amplitaq Gold™ Kit (Applied Biosystems, Thermo Fisher Scientific) according to the manufacturer's specifications. For the PCR, specific primers were designed based on the sequence of the *Rhimi-CAP*_*2b*_*R* cloned cDNA KC614697.1 [25] (Table 1). The PCR template reaction was carried out using a dilution of the synthesized cDNA (1:10), at the following temperature-cycling parameters: 1 cycle at 95 °C for 5 min; 35 cycles at 94 °C for 30 s, 55 °C for 30 s, 72 °C for 30 s; 1 cycle at 72 °C for 5 min. PCR products were cloned with the TOPO XL-2 PCR Cloning kit (Invitrogen, Thermo Fisher Scientific) and then sequenced. Correct PCR fragment insertion was confirmed for clones #4 and #6, both carrying an insert of 1537 bp, which encompasses 349 bp of the 5’-UTR end of KC614697.1, the open reading frame and 349 bp of the 3’-UTR end (Additional file 1: Data S1).

To obtain a longer 5’-UTR sequence than that originally obtained (KC614697.1), the Rapid amplification of cDNA ends (RACE) SMARTer® kit (Takara, Kusatsu, Shiga, Japan) was used, following the manufacturer’s instructions as laid down in the SMARTer® RACE 5’/3’ Kit User Manual. To synthesize the 5’-RACE-Ready first-strand cDNA, total RNA was first extracted from *R. microplus* Deutch strain whole unfed females using the same methods and kit as described above. The reaction was carried out in a total reaction volume of 20 μl containing 1 µg total RNA; the temperature-cycling parameters for cDNA synthesis were: 1 cycle at 72ºC for 3 min; 1 cycle at 42 °C for 2 min; 1 cycle at 42 °C for 90 min; and 1 cycle at 70 °C for 10 min. To extend the 5’-UTR region, a RACE PCR was run using 2.5 µl of 5’-RACE-Ready first-strand cDNA, the SMARTer® RACE 5’/3’ Kit buffers and master mix and gene-specific primers and universal and nested primers (Table 1). The temperature-cycling parameters for the RACE PCR were as follows: 5 cycles at 94 °C for 30 s, 72 °C for 3 min; 5 cycles at 94 °C for 30 s, 70 °C for 30 s, 72 °C for 3 min; 25 cycles at 94 °C for 30 s, 68 °C for 30 s, 72 °C for 3 min. The obtained 5’-UTR-RACE PCR fragment was also cloned with the TOPO XL-2 cloning kit and sequenced (Additional file 1: Data S1). This sequence was submitted to the GenBank database under accession number OP191701.

Target sequences for RNAi of 150– 250 nucleotides (nt) in length were selected from the 5’-UTR of *Rhimi-CAP*_*2b*_*R* cloned cDNA KC614697.1, [25] and from the *R. microplus* periviscerokinin receptor 5’-UTR-RACE fragment extended sequence obtained as described above (Additional file 2: Data S2). After the release of the *R. microplus* genome, further analysis became possible to test the specificity of the designed *Rhimi-CAP*_*2b*_*R* dsRNAs, to avoid potential off-target RNAi effects. To this end, for each *Rhimi-CAP*_*2b*_*R* dsRNA sequence, the algorithm BLASTn was used in searches https://www.ncbi.nlm.nih.gov/ to identify similar sequences in the genome (*R. microplus*, assembly ASM1333972v1) (Additional file 3: Data S3).

dsRNAs for the in vivo gene silencing experiments were synthesized following the manufacturer’s instructions, using the T7 RiboMAX™ Express RNAi system (Promega, Madison, WI, USA) and cDNA from whole unfed females as template. Oligonucleotide primer sequences used for dsRNA synthesis (Table 1) were designed using the Oligo v6.71 Primer analysis software www.oligo.net (Molecular Biology Insights, Inc., DBA Oligo, Inc., Colorado Springs, CO, USA), and adapted by the addition of the T7 polymerase recognition sequence, as specified by the manufacturer for dsRNA synthesis (Table 1). The concentration of dsRNA synthesized was determined using a Nanodrop 1000 spectrophotometer using optical density A_260_/A_280_ ratios (Thermo Fisher Scientific). The dsRNA sequences for the negative (beta-lactamase) and positive [*R. microplus* beta-actin gene (*Rhimi-ACTB*)] dsRNA controls were successfully used for our recently published study [30]. The primers used for the synthesis of the control dsRNAs are listed in Table 1. The beta-lactamase gene (*BLA*) encodes a bacterial enzyme that is not present in ticks. The beta-actin gene (*ACTB*) encodes for a protein involved in cell motility, structure and integrity [61].

#### Experimental design and microinjection of ticks

For the RNAi experiments, unfed CFT adult females were collected at between 1 and 5 days after molt from nymphs and used for each treatment. Ticks from each treatment were held in round, cotton sleeves that were glued to the backs of calves [29, 62]. Each sleeve contained 35–40 females and 15–25 males to allow mating. The sleeves were distributed symmetrically on both sides of the animal, and each animal was considered to be a randomized block. Five calves were used for these RNAi experiments.

Five to seven independent RNAi experiments were performed per treatment, over three different dates: January 2018, December 2019 and July 2021. Each replicate consisted of dsRNA-injected ticks and non-injected ticks (as the negative control). Female ticks were microinjected with dsRNA specific for each treatment: (i) dsRNAs for *Rhimi-CAP*_*2b*_*R*, designated ds680-805 (196 bp), ds956-1109 (172 bp) and ds1102-1200 (132 bp); (ii)* Rhimi-ACTB* as positive controls (dsβ-act); and (iii) beta-lactamase as negative controls (dsβ-lac). For the first assay (January 2018), ticks were injected with 10^11^–10^12^ dsRNA molecules/tick in 0.2 µl of NF-water. For the assays conducted in December 2019 and July 2021, dsRNA microinjections consisted of 0.2 µl of a solution prepared using NF-water and dsRNAs at concentrations ranging from 5.21 to 8.27 µg/µl, with the exception of dsRNA-*Rhimi-ACTB*, whose concentration ranged from 5.6 to 9.3 µg/µl (Additional file 4: Table S1). The methods described for tick micro-injection were used in all assays and have been previously described [63]. Injected ticks were transferred and held in cotton-stoppered glass vials in the laboratory under constant temperature (25 ± 2 °C) and 95% RH for 24 h [64]. Subsequently, live (obviously motile) injected and non-injected females were transferred to the sleeves on the calf.

The delay between the second and third replicate was due to the SARS-COVID-19 pandemic causing the closure of the facilities for animal experimentation at the USDA-ARS facility, Edinburg, Texas. Experiments described herein were conducted in parallel with those described in a previous publication [30].

#### Tissue collection, RNA extraction, cDNA synthesis and qRT-PCR for evaluation of gene silencing

To evaluate *Rhimi-CAP*_*2b*_*R* silencing after dsRNA injections, three tick females per treatment and replicate were collected on the third and fifth day after being placed on the animal. Females that fed for 3 days were individually kept in 250 µl of RNAlater™ (Invitrogen) at − 80 °C until use. Whole ticks were used to test silencing at the organism level. In contrast, females that had been 5 days on the animal, were dissected under cold physiological saline and tissues were individually kept at − 80 °C until RNA isolation. RNAlater™ was used to keep each carcass (250 µl), salivary glands, Malpighian tubules and the complete female reproductive system that included ovaries, uterus, vagina and associated glands (150 µl). Synganglion was immersed directly in 50 µl of Trizol™ reagent (Invitrogen, Thermo Fisher Scienttific) to improve RNA extraction, since the synganglion is a very small tissue that becomes fully transparent in RNAlater™ and cannot be visualized for recovery. Tissues selected to analyze the silencing efficiency were those in which a relative high expression for *Rhimi-CAP*_*2b*_*R* transcript had been previously reported in *R. microplus* for partially fed females of similar age [25].

To evaluate the effect of dsRNAs through the analysis of relative gene expression, we used the protocols for total RNA extraction, cDNA synthesis and qRT-PCR analysis and the sequences of the primers described in sections RNA isolation, cDNA synthesis and quantitative reverse-transcriptase PCR analysis and Quantitative reverse-transcriptase PCR analyses for evaluation of expression and silencing.

For the first experiment, performed January 2018, only whole ticks “3 days on the animal” were analyzed to verify gene silencing of ticks that had been injected with the three *Rhimi-CAP*_*2b*_*R* dsRNAs: dsRNA680-805, ds956-1109 and ds1102-1200. The cDNA samples for non-injected ticks, beta lactamase-injected-ticks and ticks injected with each of the mentioned dsRNAs were arranged on the same plate and run in duplicate. Three such 96-well plates were run. Two of the plates were used to analyze relative expression using primers for each reference gene, *Rhimi-EF1A* and *Rhimi-RSP4*, respectively, and the third plate was used to analyze relative expression of the gene of interest, *Rhimi-CAP*_*2b*_*R*, using primers for this gene.

For each of the other four replicates, December 2019 (1 replicate) and July 2021 (3 replicates), three 96-well plates were similarly loaded with cDNA samples for qRT-PCR analyses: two of these plates were used to analyze relative expression using primers for each reference gene, *Rhimi-EF1A* and *Rhimi-RSP4*, respectively, and the third plate, was used to analyzed relative expression of the gene of interest, *Rhimi-CAP*_*2b*_*R*, using primers for this gene. All plates were prepared with the same arrangement: the specific tissue cDNAs for all treatments, that is cDNAs of non-injected ticks, *Rhimi-CAP*_*2b*_*R* dsRNA1102-1200-injected ticks and beta-lactamase dsRNA-injected ticks were loaded, as the statistical comparisons were among treatments. Each of the following tissues were analyzed: (i) whole ticks 3 days on the animal and (ii) tissues from ticks that had fed on animals for 5 days, including carcasses, female reproductive system, synganglion, salivary glands and Malpighian tubules. In sum, three plates were prepared for each tissue.

The gene normalized relative quantity (NRQ) was calculated for *Rhimi-CAP*_*2b*_*R* following the protocol described in section Quantitative reverse-transcriptase PCR analyses for evaluation of expression and silencing. The relative transcript abundance of *Rhimi-CAP*_*2b*_*R* for each set was presented as a fold-change (FC) of beta-lactamase-injected ticks (FC = 1).

#### Phenotypic data collection

To record daily tick feeding progression after ticks were placed on the animals, the sleeves were opened and ticks were photographed (Additional file 5: Table S2). The date of self-detachment from the host was recorded, and the period of time from attachment to self-detachment was designated “repletion period”. Self-detached ticks were removed, weighed, transferred to a cotton-stoppered glass vial and held at the laboratory maintenance conditions previously mentioned, with daily monitoring.

The date of the first oviposition was registered to calculate the pre-oviposition period, which extends from detachment to oviposition [60]. CFT females die after oviposition [59]; therefore, after the egg laying was finished, the dead female tick was removed from the vial and the egg masses were weighed. The egg mass incubation period was also registered, which corresponds to the period from egg laying to the first hatch. The percentage egg hatch per female (i.e. eggs that hatched per egg mass) was determined by visual estimation by the same experienced researcher (JPT) for the duration of each experiment, as previously described in [65].

Results for other phenotypic variables were recorded for each female, such as mortality, which was registered over the course of the whole experiment. The reproductive efficiency index (REI) was calculated as: (egg mass/replete female weight) × 100, according to [66]. Further, the number of females with no eggs was calculated from the number of females that survived and were placed in vials that did not lay eggs at all, and “females with no hatch,” which reflects the percentage of produced egg masses from which no egg hatching was observed at all. Finally, the overall time period from attachment to animal until hatching of the first egg for each female tick was also recorded as observation period.

As per the approved AUP that assures maintenance of animal health and taking into account an expected detachment time from the host for uninjected ticks to be between 7 and 9 days, an endpoint for the experiment of 2 weeks after tick attachment to the animals was chosen. Ticks that did not detach before this date were considered to be dead and were not included in subsequent analyses.

### Statistical analysis

Statistical analyses of phenotypic traits and qRT-PCR assays were conducted using GraphPad Prism v6 software (GraphPad Software, San Diego, CA, USA), and the results were plotted using the same software. A one-way analysis of variance (ANOVA) followed by a Tukey’s HSD test was performed to compare *Rhimi-CAP*_*2b*_*R* relative expression throughout all developmental stages, and to verify *Rhimi-CAP*_*2b*_*R* silencing in whole ticks and different female tissues. A Kruskal–Wallis ANOVA followed by a corrected Dunn’s multiple comparisons test was used to analyze phenotypic data. All results were presented as the mean ± standard error of the mean (SEM).

## Results

### *Rhimi-CAP*_*2b*_*R* expression throughout developmental stages

**Fig. 1:**
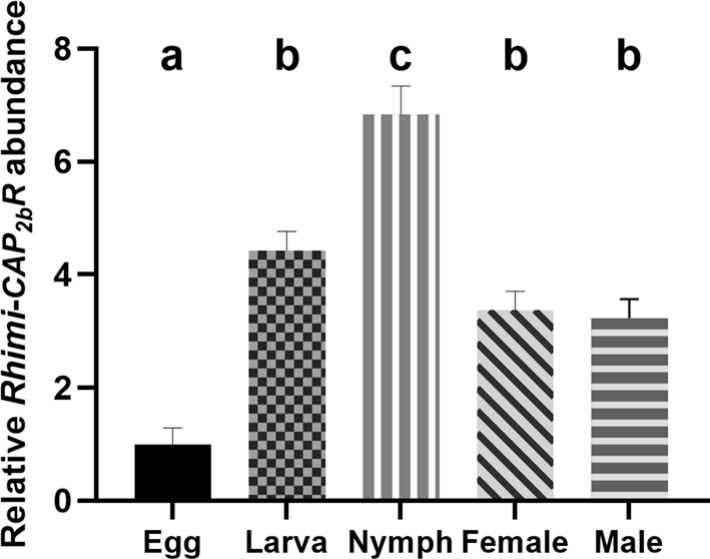
*Rhimi-CAP*_*2b*_*R* relative transcript expression throughout *Rhipicephalus microplus* developmental stages by quantitative reverse-transcriptase PCR (qRT-PCR) analyses. Stages of development were: egg masses, neolarvae, nymph, male and female. The lowest expression was observed for eggs, and the relative *Rhimi-CAP*_*2b*_*R* abundance for this stage was used as the reference ratio (calibrator) for the fold change (FC) calculations. Different lowercase letters above histogram bars indicate significant difference at *P* < 0.05, based on statistical analysis using one-way analysis of variance (ANOVA) followed by a Tukey’s multiple comparisons test. Eight (*n* = 8) biological replicates were analyzed per stage.* Rhimi-CAP*_*2b*_*R*, *Rhipicephalus* *microplus* periviscerokinin receptor

The qRT-PCR analyses detected the *Rhimi-CAP*_*2b*_*R* transcript throughout all developmental stages of *R. microplus* (Fig. 1). The lowest relative expression of *Rhimi-CAP*_*2b*_*R* was observed for eggs (Fig. 1), followed in decreasing relative expression for larvae, females and males, with *Rhimi-CAP*_*2b*_*R* relative abundance in these last three stages being similar and surpassing *Rhimi-CAP*_*2b*_*R* expression in eggs by a factor of 3.2–4.4 (*n* = 8 replicates; *P* < 0.05) (Fig. 1). The highest *Rhimi-CAP*_*2b*_*R* relative expression was observed for nymphs (Fig. 1), which surpassed *Rhimi-CAP*_*2b*_*R* expression in eggs by a factor of 6.8 (*n* = 8 replicates; *P* < 0.05) (Fig. 1). Relative abundance in eggs was used as the reference ratio (calibrator) for the FC calculation.

### *Rhimi-CAP*_*2b*_*R* silencing

**Fig. 2:**
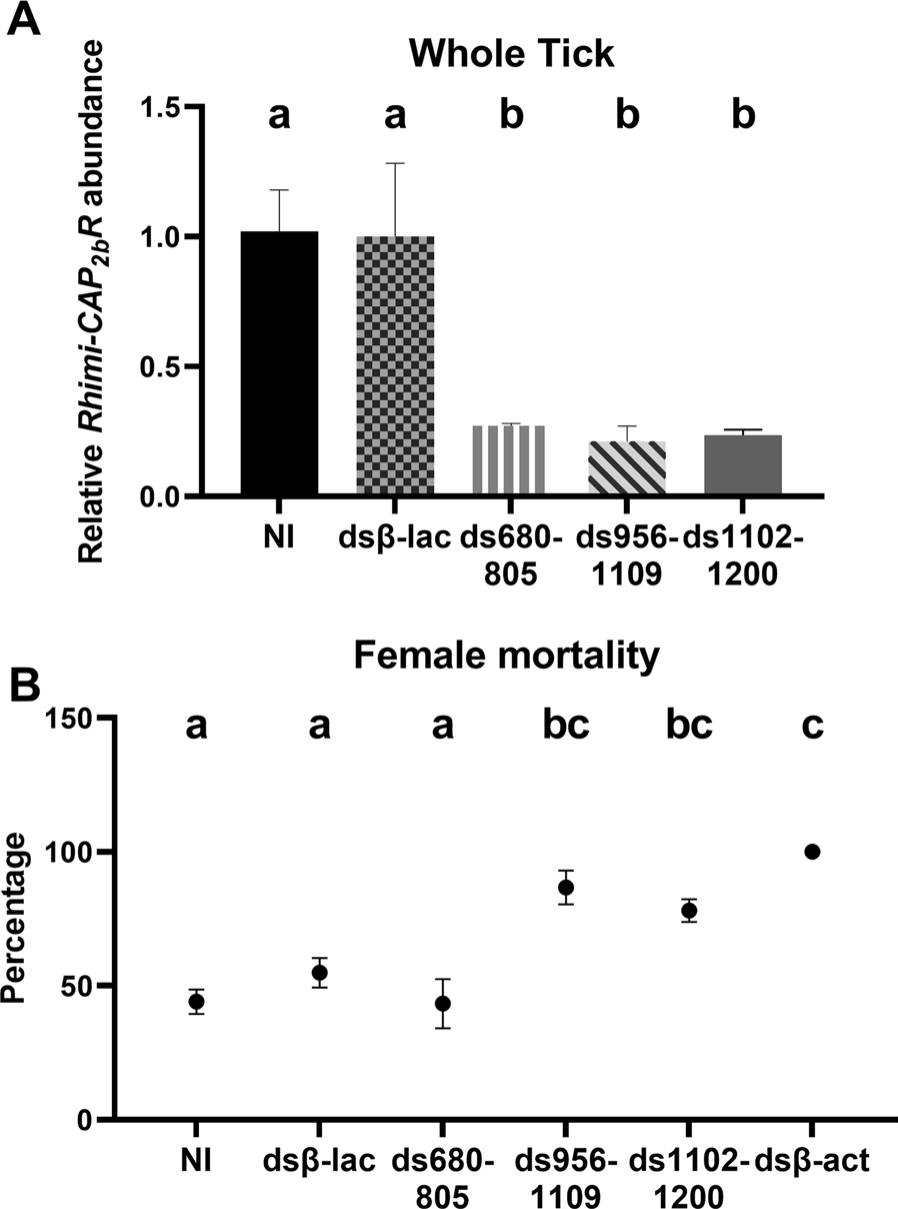
*Rhimi-CAP*_*2b*_*R* RNAi silencing using the double-stranded RNAs (dsRNAs) ds680-805, ds956-1109 and ds1102-1200.** a** *Rhimi-CAP*_*2b*_*R* silencing evaluation by qRT-PCR for whole body of partially fed females, 3 days on animals. Different lowercase letters above bars indicate significant difference at *P* < 0.05), based on statistical analysis using one-way ANOVA followed by a Tukey’s multiple comparisons test. **b** Percentage of mortality of females treated with dsRNA ds680-805, ds956-1109 or ds1102-1200 up to the assay endpoint. Different lowercase letters above graph indicate significant difference at *P* < 0.05), based on statistical analysis using one-way ANOVA followed by a Tukey’s multiple comparisons test. Between three and nine (*n* = 3–9) biological replicates were analyzed per treatment. dsβ-act, dsRNA *R. microplus* beta-actin-injected (positive control) ticks; dsβ-lac, dsRNA beta-lactamase-injected (negative control) ticks; NI, non-injected ticks

For the RNAi experiments, three *Rhimi-CAP*_*2b*_*R* dsRNAs, named ds680-805, ds956-1109 and ds1102-1200, were tested in vivo in the first assay performed on January 2018 (Fig. 2). BLASTn searches conducted to assess the risk of non-target effects only identified two sequences of ≤ 15 nt with identity to *Rhimi-CAP*_*2b*_*R* dsRNA956-1109; no such sequences were identified in the dsRNA1102-1200, which was then chosen for subsequent assays (Additional file 3: Data S3).

The present work was conducted in parallel with another study that had been published earlier [30], and the results for the negative controls [non-injected and beta-lactamase-injected females (dsβ-lac)] and positive controls (*Rhimi-ACTB* dsRNA-injected females [dsβ-act]) were shared among studies to minimize the number of calves used. Importantly, all of the RNAi treatments evaluated herein were in the same animals as the negative control treatments as a block, thereby conserving statistical validity. Silencing efficiencies of *Rhimi-CAP*_*2b*_*R* dsRNAs are reported with respect to dsβ-lac-injected ticks.

**Fig. 3:**
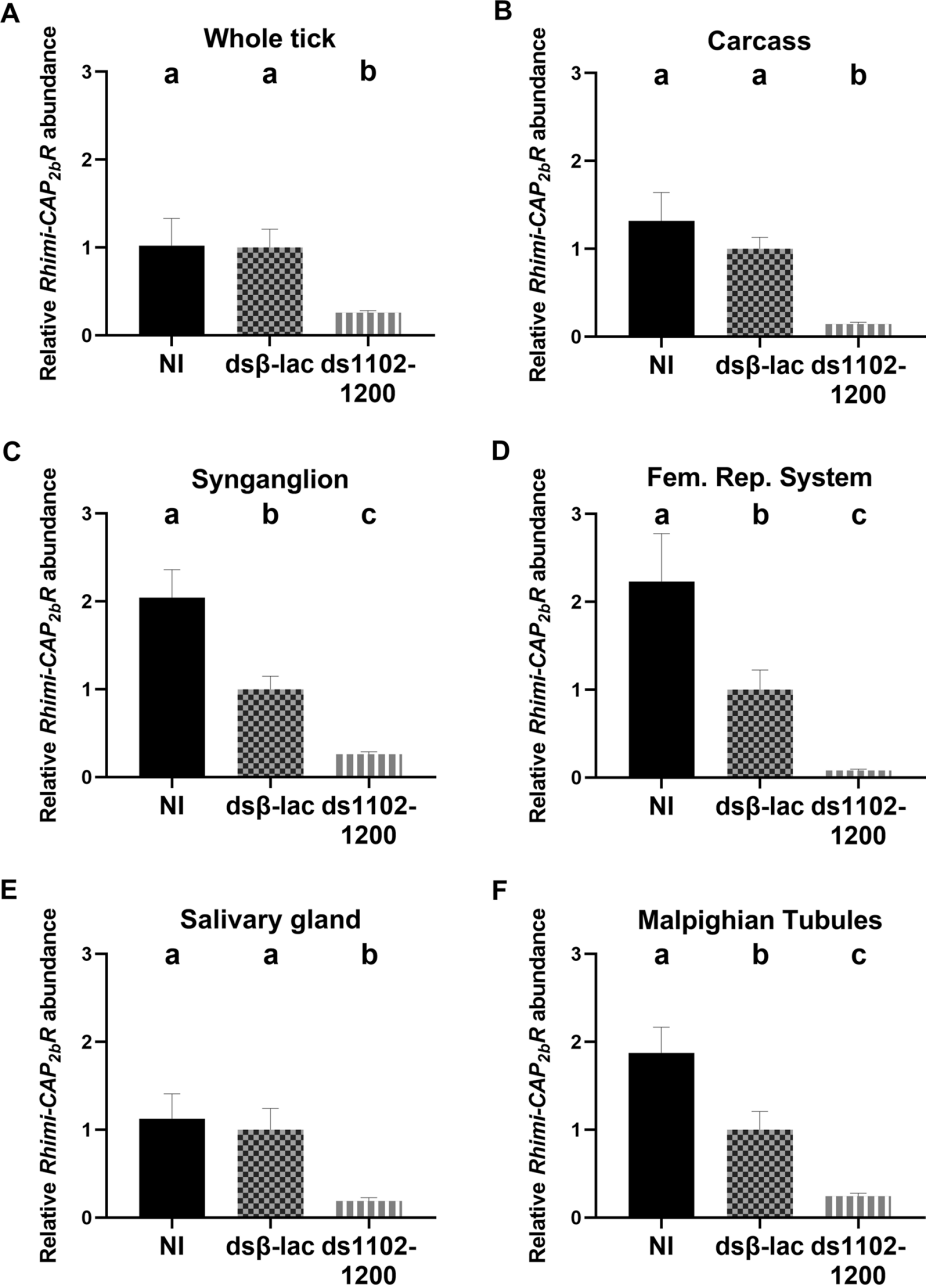
*Rhimi-CAP*_*2b*_*R* RNAi silencing using the dsRNA ds1102-1200. *Rhimi-CAP*_*2b*_*R* silencing was evaluated by qRT-PCR analyses of partially fed females from all treatments. **a** Whole tick, 3 days on animals, **b** carcass, **c** female reproductive system, **d** synganglion, **e** salivary glands, **f** Malpighian tubules. All tissues (**b**–**e**) were from female ticks 5 days on calves. Different lowercase letters above graph indicate significant difference at *P* < 0.05), based on statistical analysis using one-way ANOVA followed by a Tukey’s multiple comparisons test. Twelve (*n* = 12) biological replicates were analyzed per tissue and treatment

For the first RNAi experiment in January 2018, qRT-PCR analyses showed that the three tested dsRNAs, namely ds680-805, ds956-1109 and ds1102-1200, were equally significantly effective, producing respectively 73, 79 and 76% knockdown of *Rhimi-CAP*_*2b*_*R* mRNA in whole ticks (*n* = 3–9, *P* < 0.05; Fig. 2a). The two negative controls did not differ in terms of relative receptor transcript abundance, as expected (Fig. 2a). For subsequent experiments, performed December 2019 and July 2021, only ds1102-1200 was chosen for RNAi studies because it caused higher mortality than ds680-805, had a similar effect on mortality as ds956-1109 and, further, no identical sequence was found in the genome (Fig. 2b). Mortality after treatment with ds680-805 did not differ from the controls (Fig. 2b). qRT-PCR analyses showed that injections with ds1102-1200 caused a mRNA *Rhimi-CAP*_*2b*_*R* decrease of 74% (average for the 4 replicates) in whole ticks that were 3 days on the animal, consistent with the result of the first 2018 experiment (Fig. 3a). Females injected with ds1102-1200 that were dissected after 5 days on the animal also showed a decrease in *Rhimi-CAP*_*2b*_*R* transcript in the carcass, female reproductive system and synganglion of 85, 91, and 75%, respectively (*n* = 12, *P* < 0.05; Fig. 3b–d). These females showed a decrease in *Rhimi-CAP*_*2b*_*R* transcript expression in salivary glands and Malpighian tubules of 81% and 77%, respectively (*n* = 12, *P* < 0.05; Fig. 3e, f). All tissues from silenced females were significantly different from the negative controls in relative receptor transcript abundance (Fig. 3).

### Phenotypic changes associated to *Rhimi-CAP*_*2b*_*R* silencing

**Fig. 4:**
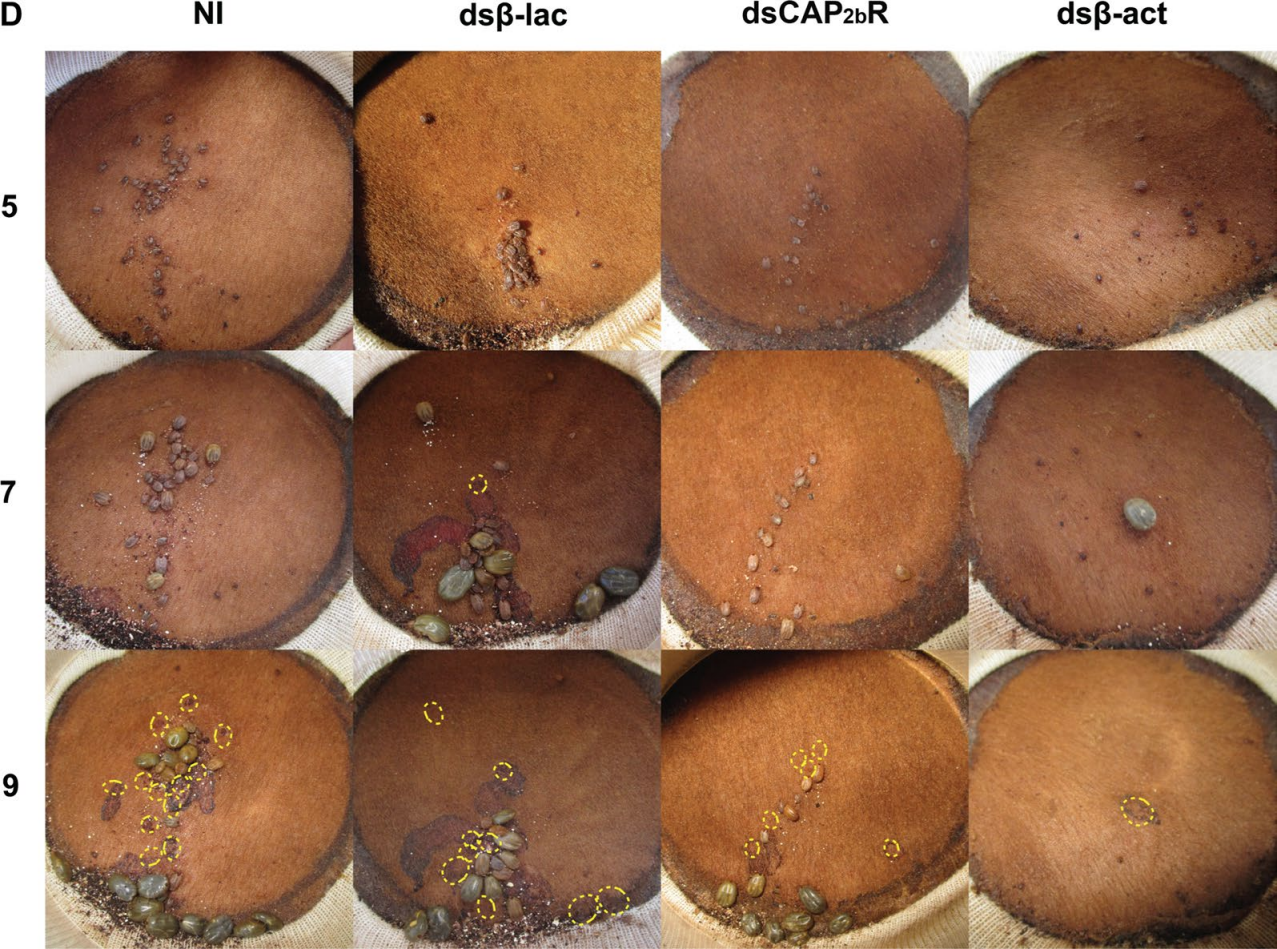
Feeding progression of *Rhimi-CAP*_*2b*_*R* silenced ticks. Photographs of open cotton sleeves showing shaved patches on cattle where the confined cattle fever tick (CFT) female ticks fed, photographed at 5, 7 and 9 days (numbers on the left side of the figure). Additional images of ticks feeding throughout the whole experiment are shown in Additional file 5: Table S2. Ticks that have already dropped from animals are highlighted with yellow circles. D, Number of days ticks feed on the animals, dsCAP_2b_R, *Rhimi-CAP*_*2b*_*R* dsRNA-injected ticks; see Fig. 2 caption for other definitions. The photographs of negative and positive controls were previously published [30] and are given here for comparison purposes

**Fig. 5:**
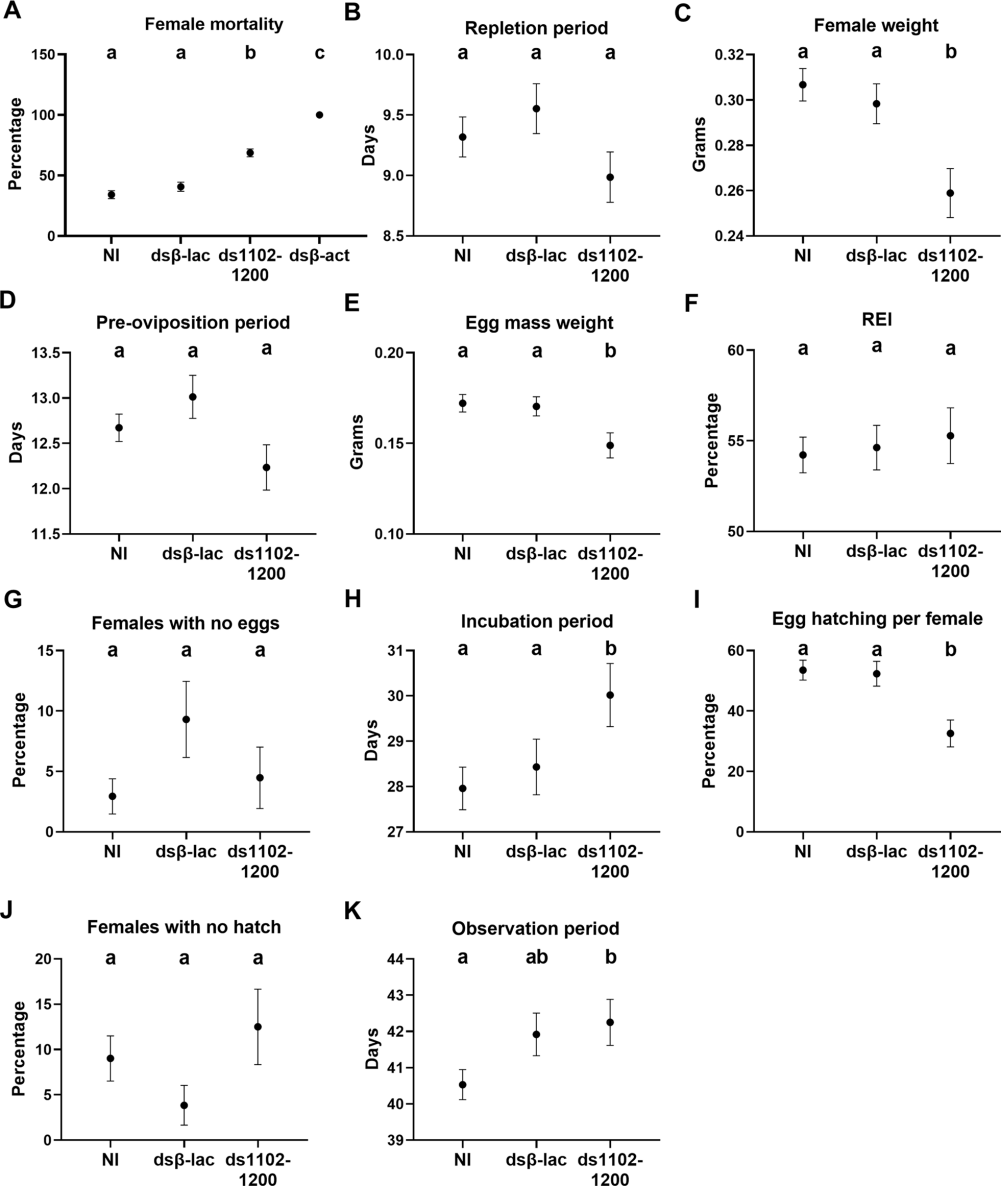
Phenotypic evaluation of CFT females after *Rhimi-CAP*_*2b*_*R* silencing, using the dsRNA ds1102-1200.** a** Percentage of female mortality, **b** duration of repletion period, **c** female weight, **d** duration of pre-oviposition period, **e** egg mass weight, **f** reproductive efficiency index (REI), which reflects the conversion of the blood intake to egg production, **g** females with no eggs, which represents females that showed no oviposition at all, **h** duration of egg incubation period, **i** percentage of egg hatching per female, **j** females with no hatch, reflecting the percentage of females, whose egg mass did not show hatching at all, **k** total observation period, from tick attachment to the animals until hatching of the first egg for each female. Negative controls: NI (non-injected ticks), dsβ-lac (dsβ-lac-injected ticks), ds1102-1200 (*Rhimi-CAP*_*2b*_*R* dsRNA-injected ticks). Positive control: dsβ-act (dsβ-act-injected ticks). Treatments sharing the same lowercase letter were not significantly different from each other at *P* < 0.05 based on the Kruskal–Wallis test followed by the corrected Dunn’s multiple comparisons test

After *Rhimi-CAP*_*2b*_*R* silencing was verified by qRT-PCR analyses (Figs. 2a, 3), the phenotypic traits of the ticks under each treatment were evaluated, and results are summarized in Figs. 4 and 5. Tick pictures representing each treatment effect after 5, 7 and 9 days on the calves are shown in Fig. 4. These images reflect the RNAi effects on the phenotypic traits quantitatively evaluated in Fig. 5, and provide further visual evidence of the differential feeding progression among treatments. Many of the non-injected ticks (NI) and dsβ-lac-injected ticks fed to repletion between days 5 and 7, and by day 9 most of them had dropped from the animal (see vacant, yellow circles in Fig. 4). These records accurately reflect the significant *Rhimi-CAP*_*2b*_*R* dsRNA treatment effect in reducing female weight relative to the negative controls by the endpoint of the experiment, but these size differences resulted under a similar repletion period (Figs. 4, 5b, c). A detailed record of ticks feeding progression on calves is shown in Additional file 5: Table S2.

For the January 2018 RNAi experiment, female ticks injected with ds956-1109 and ds1102-1200 showed a significant increase in net mortality relative to the dsβ-lac-injected ticks (negative controls) of 31.79% and 23.25%, respectively (*n* = 30–92; *P* < 0.05), (Fig. 2b). Non-injected ticks and dsβ-lac-injected ticks had lower and similar mortalities (40% and 55%, respectively) than the *Rhimi-CAP*_*2b*_*R* dsRNA-treated ticks (Fig. 2b). For the December 2019 and July 2021 RNAi experiments, where only ds1102-1200 was evaluated, *Rhimi-CAP*_*2b*_*R*-silenced females showed a significant increased net mortality of 28% with respect to the dsβ-lac-injected ticks (*n* = 140–216; *P* < 0.05) (Fig. 5a). Similarly, as found earlier, the negative control ticks had a similar and lower mortality than receptor-silenced ticks, 34% for non-injected ticks and 40% for dsβ-lac-injected ticks (Fig. 5a).

For the December 2019 and July 2021 RNAi experiments, additional phenotypic traits were analyzed. *Rhimi-CAP*_*2b*_*R*-silenced females showed a significant decrease in net weight of 13% (*n* = 75–135; *P* < 0.05) (Fig. 5c) and significant decrease in net weight of their egg masses of 15% (*n* = 65–131; *P* < 0.05) (Fig. 5e), both relative to dsβ-lac-injected females. The egg incubation period was significantly longer by 1.6 days (*n* = 59–120; *P* < 0.05) relative to dsβ-lac-injected females (Fig. 5h), and significant differences were not detected between the negative control treatments for both traits (Fig. 5c, e, h). However, for the overall observation period, encompassing the female feeding period until the day of first egg hatching, there was no difference between *Rhimi-CAP*_*2b*_*R*-silenced ticks and dsβ-lac-injected females (*n* = 56–120; *P* > 0.05) (Fig. 5k).

The egg hatching was also affected by *Rhimi-CAP*_*2b*_*R* silencing, and females injected with ds1102-1200 showed a net decrease of 20% egg hatching (*n* = 58–108; *P* < 0.05) with respect to dsβ-lac-injected females (Fig. 5i). Similarly, there were no differences between the negative control treatments for both traits.

Among the traits analyzed, there were no other phenotypic changes after *Rhimi-CAP*_*2b*_*R* silencing, in comparison to the negative controls. The repletion (*n* = 71–135; *P* > 0.05) and pre-oviposition (*n* = 64–131; *P* > 0.05) periods showed no difference between *Rhimi-CAP*_*2b*_*R*-silenced ticks and negative controls (Fig. 5b, d). The REI, which reflects the percentage of conversion of female weight to egg mass (*n* = 64–131; *P* > 0.05), showed no difference between *Rhimi-CAP*_*2b*_*R*-silenced ticks and negative controls (Fig. 5f). Regarding the number of females with no eggs (live females that did not lay), no significant statistical differences were observed (*n* = 67–136; *P* > 0.05) among the treatments (Fig. 5g). Finally, the number of egg masses that showed no hatch at all (females with no hatch) (*n* = 64–133; *P* > 0.05) was not statistically different between *Rhimi-CAP*_*2b*_*R*-silenced ticks and negative controls (Fig. 5j).

## Discussion

Periviscerokinins (CAP_2b_/PVKs) are neuropeptides that elicit myotropic activity and diuretic control in insects [42–45]. In ticks, only the myotropic activity of the CAP_2b_/PVKs has been confirmed to date [54]. In the CFT, the *CAP*_*2b*_*/PVK* transcript receptor has been detected in the synganglion, salivary gland and Malpighian tubules and in the reproductive system of the female ticks [25]. We detected *CAP*_*2b*_*/PVK* receptor transcript expression in whole ticks in all developmental stages and in eggs, fulfilling the requirement of a broad developmental expression for a potential suitable target for tick control (Fig. 1). This expression pattern in ticks agrees with previous observations in insects, as transcript expression for the *CAP*_*2b*_*/PVK* receptor was observed in eggs of *Halyomorpha halys* [67] and *Bombyx mori* [68]. Also, expression of the CAP_2b_/PVK peptide precursor transcript was detected in eggs of *H. halys* [69], *Solenopsis invicta* [70] and *Locusta migratoria* [71]. One potential role of the periviscerokinin (CAP_2b_/PVKs) system in embryos of ticks could be control of myotropic activity, since muscle contraction was observed in embryos of the coleopteran insect *Callosobruchus maculatus* [72], but no peptides associated with the regulation of this process have been identified to date. Another known function of the CAP_2b_/PVK system in insects is diuretic; however, Malpighian tubules are under a process of cell proliferation and differentiation during embryonic development, and these are not fully functional until the larval stage (see, for example, [73, 74]); therefore, a function of the CAP_2b_/PVK receptor in embryo Malpighian tubules is less likely.

Here, we used a proven RNAi protocol for the silencing of the CFT *CAP*_*2b*_*/PVK* receptor (*Rhimi-CAP*_*2b*_*R*) to investigate the CAP_2b_/PVK signaling system in this tick species. This protocol was previously used successfully to silence two other tick GPCRs, namely the kinin and pyrokinin receptors [29, 30]. dsRNAs ranging from 132 to 196 bp were designed targeting the 5’-UTR region, since there is evidence of the advantage of choosing those regions over the coding sequence region, leading to higher efficacy and efficiency [75]. Possible off-target effects of the chosen *Rhimi-CAP*_*2b*_*R* dsRNAs are highly unlikely because none was found for dsRNA1102-1200 and the maximum two contiguous identical sequences in *Rhimi-CAP*_*2b*_*R* dsRNA956-1109 were 15 bp (Additional file 3: Data S3). These alignments showed that the similarities between the compared sequences were not enough to trigger off-target effects, according to previous studies that concluded that 19–21 nt of a contiguous identical sequence are required to produce significant silencing activity [75, 76]. Positive control ticks silenced for beta-actin were used in this experiment because they represent an extreme phenotypic and physiological effect of RNAi which allows relative comparisons of effects of silencing of different GPCRs against this extreme phenotype (see Figs. 2b, 4, 5a).

We will speculate next on the potential roles that the CAP_2b_/PVK system may have in ticks, as suggested by qRT-PCR analyses, observed phenotypes upon silencing the *Rhimi-CAP*_*2b*_*R* and the known PRX-amide peptide functions in insects. The following hypotheses will require testing in ticks.

After the *Rhimi-CAP*_*2b*_*R* silencing, we observed a decreased female weight (Fig. 5c), as evidenced by a smaller size in photgraphs documenting the experiment (Fig. 4), which was obvious in comparisons on day 7 in dsβ-lac- versus* Rhimi-CAP*_*2b*_*R* dsRNA-injected females. Our results suggest a direct or indirect role of the CAP_2b_/PVK signaling system in the regulation of female feeding and growth in *R. microplus*. However, no differences were observed on the duration of the repletion period between treated and control ticks (Fig. 5b). These results differ from previous observations on RNAi of GPCRs associated with female feeding in *R. microplus*, in which after silencing of the kinin and pyrokinin receptors, there was a simultaneous decrease in female weight and extension of the repletion period [29, 30].

Ticks are pool feeders, and the feeding process involves penetration of the host skin by the hypostome, retraction of the cheliceral shafts and lateral movement of the cheliceral digits to introduce the feeding apparatus inside of the host [77]. Then, the blood is sucked into the food canal and pharynx by contraction of the dilator muscles of the pharynx, and relaxation of these muscles allows the blood to pass through the esophagus into the midgut [78]. Precise contraction of the feeding-related muscles represents a key aspect of this process. Recently, we demonstrated the myotropic activity of pyrokinins (PKs) in feeding-related tissues of two tick species, *R. sanguineus* and *I. scapularis* [32]. Further, myotropic activity was confirmed in the hindgut of *I. scapularis* for CAP_2b_/PVKs [54] utilizing the CAPA-PVK1 peptide PALIPFPRVa, as reported in [50]. The cDNA we previously cloned from *R. microplus* includes PKs and two CAP_2b_/PVKs [32, 52]; this co-expression could suggest a role of the CAP_2b_/PVK signaling system on the myotropic activity of feeding-related tissues, as shown for PKs; however, the myotropic activity of CAP_2b_/PVKs on feeding-related tissues has not yet been investigated. Alternatively or simultaneously, the silencing of the *Rhimi-CAP*_*2b*_*R* could have diminished hindgut contractions, negatively affecting excretion [54].

Neupert et al*.* [50] co-immunolocalized kinin (LK) and CAP_2b_/PVK in several neurons in the synganglion of ticks *I. ricinus* and *R. microplus*. The cheliceral ganglion integrates the supraesophageal region of the synganglion. The cheliceral nerve projects from the cheliceral ganglion and innervates the chelicerae [79]. Only LK-positive immunostaining was observed in the T1 neurons located in the anterior cheliceral ganglion [50]. Further, nerves from the supraesophageal area innervate salivary glands, the pharynx and the esophagous. Neuronal arborizations showing CAP_2b_/PVK-positive staining were observed in the supraesophageal region [50]. Consequently, the CAP_2b_/PVK signaling system could be associated with the feeding process in conjunction with the LK system. The *CAP*_*2b*_*/PVK* receptor transcript has been found to be expressed in the salivary glands of *R. microplus* [25]; the relative transcript expression level was similar to that in other tissues, such as the synganglion, ovary and Malpighian tubules [25]. Tick salivary secretions are injected into the feeding site to modulate the host's hemostasis and immune response, allowing for a long feeding period [80]. This could suggest that the CAP_2b_/PVK signaling system could be associated with the control of salivary gland function.

After the *Rhimi-CAP*_*2b*_*R* silencing, the female reproductive output was affected through a decreased egg mass weight (Fig. 5e), a longer egg incubation period (Fig. 5h) and a lower percentage of egg hatch, in comparison to the dsβ-lac-injected ticks (Fig. 5i). In ticks, the magnitude of female engorgement, in terms of quantity and quality of food intake, directly affects the development of the ovaries, egg maturation and the number of eggs produced [79]. The decrease in egg mass weight after *Rhimi-CAP*_*2b*_*R* silencing could be associated to a decreased blood intake (Figs. 4, 5c), but also with a reduction of CAP_2b_/PVK activity in the female reproductive tissues themselves, since *Rhimi-CAP*_*2b*_*R* transcript expression was observed in these tissues. Defects in feeding of silenced females, if resulting in a decreased heme acquisition, could explain a delayed incubation period and reduced egg hatch.

Heme and iron homeostasis are essential for normal tick feeding and reproduction [81–83]. In *R. microplus*, heme lipoproteins are present in the ovary [82] but also in the salivary glands [84], suggesting roles in reproduction and feeding. In *R. sanguineus*, silencing of ferritin 1 (FER1) and 2 (FER2), two proteins involved in iron metabolism, is associated with defects in feeding [81, 83] and egg hatching [81], respectively. Further, [81] found that depletion of FER2 from the tick hemolymph leads to a loss of FER1 expression in the salivary glands and ovaries after a blood ingestion. Moreover, vitellogenin (Vg) is responsible for heme binding and transport to the ovaries in hematophagous arthropods, including ticks [85, 86]. Consequently, the decrease in the reproductive performance observed here after *Rhimi-CAP*_*2b*_*R* silencing (Figs. 5E, H and I), could be associated to defects in both, vitellogenesis and normal heme/iron homeostasis.

In *R. microplus*, *CAP*_*2b*_*/PVK* receptor transcript expression was found in the female reproductive tissues [25]. In the coleopteran *T. castaneum*, silencing of *CAP*_*2b*_*/PVK* receptor led to a significant reduction in Vg accumulation in developing oocytes and a reduction of ~ 75% in egg production [87]. Further, in the whitefly *Bemisia tabaci* egg production was significantly reduced after CAP_2b_/PVK receptor silencing [88]. In ticks, Hussein et al. [86] observed that silencing of VgR in *R. microplus*, was followed by decreased egg production, delayed embryonic development and reduced egg hatching. In the present study, the same three phenotypic results were observed for *R. microplus* females after *Rhimi-CAP*_*2b*_*R* silencing (Fig. 5e, h, i). Based on these insect reports and our RNAi observations in CFT, it would be worthy to investigate the relationship between tick Cap2b/PVKR and vitellogenesis regulation in ticks.

The C-terminal sequence **WFG**PRXa (X = L, I, M or V) is a highly conserved motif for the diapause hormones (DHs) in insects (with exceptions for some Paleoptera and Apterygota), and DHs are coded by two different genes: the *CAPA* gene encodes DH-1 and the *PK/PBAN* gene, DH-2 [34]. In Lepidoptera, DH initiates embryonic diapause in *Bombyx mori* [89, 90] and breaks pupal diapause in *Heliothis* and *Helicoverpa* spp. [91–94]. However, none of the predicted CAPA/PK peptides annotated for ticks, including *R. microplus*, carries the conserved motif mentioned above, specifically the **WFG** motif, as the PRXa C-terminus is conserved in all family members [32]. Several types of diapause have been observed in Prostriata and Metastriata ticks [95–97], including a delay of oogenesis in engorged females and a delay in the onset of embryogenesis in eggs [95]. Only a few studies have been conducted on hormonal control of diapause in ticks. In *R. sanguineus* and *Dermacentor albipictus* (Acari: Ixodidae), a breaking of larval diapause was observed after ecdysteroid treatment [98, 99]. In the present study, we observed a delayed embryonic development after *Rhimi-CAP*_*2b*_*R* silencing (Fig. 5h); therefore, it would be important to determine the relationship between the CAP_2b_/PVK signaling system and ecdysteroidogenesis, as it may appear that the CAP_2b_/PVK receptor loss of function could decrease ecdysteroids.

In *D. melanogaster,* CAP_2b_/PVK silencing is associated with increased mortality under cold and/or desiccation stress conditions [100]. Silencing of the CAP_2b_/PVK receptor in the whitefly *Bemisia tabaci* led to a high mortality of approximately 30% [88], similar to the net 28% increase in mortality obtained in our study for *Rhimi-CAP*_*2b*_*R* silencing (Fig. 5a). These authors of the *Bemisia tabaci* study [88] suggested that mortality could be associated with a deregulation of the water/ionic balance. CAP_2b_/PVK transcript expression was detected in Malpighian tubules in *R. microplus* [27, 28], and myotropic activity of periviscerokinin was shown in the hindgut [54]; and in the present study the receptor transcript was significantly reduced by RNAi in these tissues (Fig. 3f). Thus, a CAP_2b_/PVK role in fluid homeostasis regulation in ticks can also be suggested.

## Conclusions

Our results suggest that the CAP_2b_/PVK signaling system is significant in tick physiology, since loss of CAP_2b_/PVK receptor transcript expression was associated with reduced female weight, defects in reproduction and increased female mortality. These results indicate that the CAP_2b_/PVK system does not have a redundant function in ticks, or at least not a fully redundant function as there were significant effects. We had previously functionally characterized the CAP_2b_/PVK receptor in *R. microplus* [27]. Recently, after pyrokinin receptor and leukokinin receptor silencing, we observed increased female mortality and decreased weight of eggs and females, similar to our observations in the present study; however, among the three, only *Rhimi-CAP*_*2b*_*R* silencing caused a 20% reduction in egg hatching [29, 30]. Importantly, *Rhimi-CAP*_*2b*_*R* silencing was associated with a decrease in egg production which, combined with the reduction in egg hatching, will have a critical impact in reducing the numbers of the next generation.

 In summary, our results support that the PRX-amide neuropeptide family may have pleiotropic functions in ticks and, accordingly, antagonists of these signaling systems could be explored for tick control.

## Supplementary Information

**Additional file 1: Data S1. **Nucleotide sequences of the *Rhimi-CAP*_*2b*_*R* (KC614697.1), two *Rhimi-CAP*_*2b*_*R* clones (clones #4 and #6) both identical to the original KC614697.1 cDNA, and a 5’-RACE PCR sequence obtained to extend the 5’-UTR region of the* R. microplus* periviscerokinin receptor. An alignment between the* R. microplus* genome (isolate Rmic-2018 chromosome 3, ASM1333972v1, NC_051167.1) and the* R. microplus* periviscerokinin receptor extended RACE-5’-UTR fragment (Accession number OP191701) is provided.

**Additional file 2: Data S2. **Nucleotide sequences of the *Rhimi-CAP*_*2b*_*R* (KC614697.1) and the extended *Rhimi-CAP*_*2b*_*R* 5’-UTR sequence. The dsRNAs used for *Rhimi-CAP*_*2b*_*R* silencing are displayed on these sequences. Further, an EMBO-Clustal Omega 1.2.4 multiple sequence alignment between KC614697.1 sequence and* R. microplus* periviscerokinin receptor RACE 5’-UTR fragment (R-5’CAP2bR) (Accession number OP191701) is provided.

**Additional file 3: Data S3. **NCBI-BLASTn searches to check for possible off-target effects of the *Rhimi-CAP*_*2b*_*R* dsRNA sequences. Only two short identical sequences to ds956-1109 were found to be ≤ 15 nt in length, which is not sufficient to cause off-target RNAi effects.

**Additional file 4: Table S1. **dsRNA treatments and respective concentrations used in each replicate of the RNAi experiment.

**Additional file 5: Table S2. **Pictures of ticks showing feeding progression from days 6 to 11 (December 2019), or from days 5 to 14 (July 2021) for all treatments.

## Data Availability

The data used and/or analyzed during the study are available from corresponding author on reasonable request. All relevant data are in the manuscript.

## Abbreviations

AUP: Animal use protocols
CAP_2b_/PVKs: Periviscerokinins
cDNA: Complementary deoxyribonucleic acid
CFTRL: Cattle fever tick research laboratory
CFT: Cattle fever tick
DH-1: Diapause hormone 1
DH-2: Diapause hormone 2
DNAse 1: Deoxyribonuclease 1
FC: Fold-change
GPCR: G-protein-coupled receptor
NF-water: Nuclease-free water
nt: Nucleotides
PKs: Pyrokinins
PK/PBAN/DH: Pyrokinin/pheromone biosynthesis activating neuropeptide/diapause hormone
PKR: Pyrokinin receptor
PSOs: Perisympathetic organs
REI: Reproductive efficiency index
RH: Relative humidity
RNAi: RNA interference
SEM: Standard error of the mean
qRT-PCR: Quantitative reverse-transcriptase PCR
USDA-ARS: US Department of Agriculture-Agricultural Research Service
UTR: Untranslated region
