# Supplementary material for: Periviscerokinin (Cap2b; CAPA) receptor silencing in females of Rhipicephalus microplus reduces survival, weight and reproductive output
Source: Parasit Vectors. 2022 Oct 6;15:359. doi: 10.1186/s13071-022-05457-7 (PMC9535995; doi:10.1186/s13071-022-05457-7)
Supplement: Supplementary file 1 — Additional file 1: Data S1. Nucleotide sequences of the Rhimi-CAP2bR (KC614697.1), two Rhimi-CAP2bR clones (clones #4 and #6) both identical to the original KC614697.1 cDNA, and a 5’-RACE PCR sequence obtained to extend the 5’-UTR region of the R. microplus periviscerokinin receptor. An alignment between the R. microplus genome (isolate Rmic-2018 chromosome 3, ASM1333972v1, NC_051167.1) and the R. microplus periviscerokinin receptor extended RACE-5’-UTR fragment (Accession number OP191701) is provided. [file 13071_2022_5457_MOESM1_ESM.docx]

Start and stop codons are highlighted in **underlined bold letters**

**Primers positions**

**RmPerivis-200U19 - Rhimi-CAP2bR RT-PCR**

**RmPerivis-1716L21 - Rhimi-CAP2bR RT-PCR**

**RmPerivis-388L18 - 5’-RACE-PCR**

**RmPerivis-195L20 - 5’-RACE-PCR**

**Sequences**

***Rhimi-CAP_2b_R* (GenBank KC614697.1) 1759 bp**

ACATGGGTGATCTAAGCCTAATAGTGGGACCTCACTTAGCAATCACCACTTAAGTGAAGGTTTCGCTTGAAGAATCTGTGAGTTATGTGAAACTGTCGTCAGGGACGTCAAGCCGAAGAATATCTCACCATACTCTGTGGTCACCTGTGTATGTCACTAAGAAGAAACTACCAAGAAATAGCTGCGCCTTCGCA**GGACTATCTGCTGTGCCGAC**ACTGTGAAGGGTGTCAGGTTGTGATGCGTTGGCTCGGTCACATCGGGATCACGGTGC**ATG**GAGAACACTGAAATAGGACAGATGATTATTCTTGTGGTGCTGTGAGTTCCGTGCATCGAGGACAGTGCACTGTGATGGAGAACGTAGCTGTGGCAGCCGTGAGTTCGTTGGAGGACAACGCCACCATACGGGAGTACTTGGAACTCAGGCTGGGACCCCAGCACATACTGCTGCCAATCGTGATCCCACTCACGGTACTCTACGTGGTGGTGTTCGTGAGCGGCGTGGTGGGCAACGTCACGGTATGCCTGGTGATTGCGCGCAATTCTCACTTCCAGACACCCACCAACTACTACCTCTTCTCGCTAGCCATCTCGGATCTGCTCATACTCGTCTTCGGCCTGCCCAACGACCTGAAGCTCTACTGGCAACAGTACCCATGGCGACTGGGCGAGGCTCTGTGTCGCTTCAGGGCCTTGGTGGCAGAAGCCACTTCGTACGCCTCAGTATTGACCATCGTGGCGTTCACCGCAGAACGTTACGTGGCCATCTATCACCCTCTCTTCCTACAGACCACATCAAGCCTGACTCGGGCCGTGCGCATCATCGCCATCATCTGGGTGGTGTCGCTGGTGAGCGCCATACCGTTCGCCATCTACACTCGGGTCAACTTCGTTGACTTCCCTGTTGGCTCGGGTCGCGTGGTGCCCGAGTCAGCTTTCTGTGCCCTACCGATGGACACGACTGCGGTCAGCCTTCCCCTGCTGCAGTGCTCGACGTTCGCCTTCTTCTGCCTTCCCATGACAGTCATAGCAGTGCTGTACCTCAAGATAGGAATGCGGTTAAGGAGCCAGCCAGGACCCGGGCAGCAGGGGAGGCACCAGCGCAGACCAGTGCACAGAATGCTGGTGGCGGTGGTCATAGCGTTCTTCGTCTGCTGGGCTCCGTTCCACACCCAGCGCCTTCTGGTGGTGTACGTCAGCCCCACCCAATGGACTACGGGCCTACGCACTCTCAACGAAGTGCTCTACTACACAGCCGGGTGTCTCTACTACTTCAGTGCCACAATTAACCCGATACTCTACTCGCTGATGTCCGTCAAGTACCGAGAGGCATTTCGCGACGCTCTCTGCACCCTGTCCAAGAATAAGCAGCGGCTCTCAGCTGGAGATTTCGATGCCGGTGCCACCGTAGTTATCGTTGGTGCCGGCCACTCTATATTGGACAACACGAGGCTGAGCACGCTAAAGCCATACTCGATCGTCCAGCGCGTTGACACCGATGAGGCAAACGTGGACACGGCCGCGGACATAGCGATGCGGGCCCTCATCATCCAGACGGTGCTCCGCGTTAGGCCTAACCAGGAACCGCCATTGACGCCAAGTATGAGCGAAGCTGACGATAAGGAGAAAATTCCCGTCAAGGAGATCTCCTCCAGTGACAAGAGCCCTCTGCCTTCGGAGACGGTGGTG**TGA**CTCTCCACCTTGGTGACTCTTGTAGGCATAAACGCCATACCCTGTCCAAAAAAAAAAAAAAAAAAAAAAA

***R. microplus* Periviserokinin receptor sequencing.**

Two clones were sequenced (clones #4 and #6) and a 5’ RACE PCR, conducted to extend the 5’-UTR region of the *R. microplus* periviscerokinin receptor.

**Primers used**

**RmPerivis-200U19 - Rhimi-CAP2bR RT-PCR**

**RmPerivis-1716L21 - Rhimi-CAP2bR RT-PCR**

**Clones**

**Clone #4**

ATCTGCTGTGCCGACACTGTGAAGGGTGTCAGGTTGTGATGCGTAGGCTCGGTCACATCGTGATCACAGTGCATCGAGAACACTGAAATAGGGCAGATGATTATCCTTGCGGTGCTGTGAGTTCCGTGCATCGAGGACAGTGCACTGTGATGGAGAACGTAGCGGTGGCAGCCGTGAGTTCGTTGGAGGACAACGCCACCATACGGGAGTACTTGGAACTCAGGCTGGGACCCCAGCACATACTGCTGCCAATCGTGATCCCACTCACGGTTCTCTACGTGGTGGTGTTCGTGAGCGGCGTGGTGGGCAACGTCACGGTGTGCCTGGTGATTGCGCGCAACTCTCACTTCCAGACACCCACCAACTACTACCTCTTCTCGCTAGCCATCTCGGATCTGCTCATACTCGTCTTCGGTCTGCCCAATGACCTGAAGCTCTACTGGCAACAGTACCCATGGCGACTGGGCGAAGCTCTGTGTCGCTTCAGGGCCTTGGTGGCAGAAGCCACATCGTACGCCTCAGTATTGACCATCGTGGCGTTCACTGCTGAACGTTACGTGGCCATCTATCATCCTCTATTCCTACAGACCACATCGAGCCTGACTCGGGCCGTGCGCATCATCGCCATCATCTGGGTGGTGTCGCTGGTGAGCGTCATACCGTTCGCCATCTACACTCGGGTCAACTTCGTGGACTTCCCTGTTGGCTCGGGTCGCGTGGTGCCCGAGTCAGCTTTCTGTGCCCTACCGATGGACACGACTGCGGTCAGCCTTCCCCTGCTGCAGTGCTCGACGTTCGCCTTCTTCTGCCTTCCCATGACAGTCATAGCAGTGCTGTACCTCAAGATAGGAATGCGGTTAAGGAGCCAGCCAGGACCCGGGCAGCAGGGGAGGCACCAGCGCAGACCAGTGCACAGAATGCTAGTGGCTGTGGTCATAGCGTTCTTCGTCTGCTGGGCTCCGTTCCACACCCAGCGCCTTCTGGTGGTGTACGTCAGCCCCACCCAATGGACTACGGGCCTACGCACTCTCAACGAAGTGCTCTACTACACAGCTGGGTGTCTCTACTACTTCAGTGCCACAATTAACCCGATACTCTACTCGCTGATGTCCGTCAAGTACCGAGAGGCATTTCGCGACGCCCTCTGCACCCTGTCCAAGAATAAGCAGCGGCTCTCAGCTGGAGATTTCGATGCCGGTGCCACCGTAGTTATCGTCGGTGCTGGCCACTCTATATTGGACAACACAAGGCTGAGCACGCTAAAGCCATACTCGATCGTCCAGCGCGTTGACACCGAGGAGGCTAACGTGGACACCGCCGCGGACATAGCGATGCGGGCCCTCATCATCCAGACGGTGCTCCGCGTTAGGCCTAACCAGGAACCGCCACTGACGCCAAGTATGAGCGAAGCTGATGATAAGGAGAAAATTCCCATCAAAGAGATCTCCTCCAGTGACAAGAGCCCCCTGCCTTCGGAGACGGTGGTGTGACTCTCCACCTTGGTGACTCTTGTAGGCATAAACGCCATACCCTGTTC

**Clone #6** (has only one nucleotide difference in comparison to Clone #4, yellow highlight)

ATCTGCTGTGCCGACACTGTGAAGGGTGTCAGGTTGTGATGCGTAGGCTCGGTCACATCGTGATCACAGTGCATCGAGAACACTGAAATAGGGCAGATGATTATCCTTGCGGTGCTGTGAGTTCCGTGCATCGAGGACAGTGCACTGTGATGGAGAACGTAGCGGTGGCAGCCGTGAGTTCGTTGGAGGACAACGCCACCATACGGGAGTACTTGGAACTCAGGCTGGGACCCCAGCACATACTGCTGCCAATCGTGATCCCACTCACGGTTCTCTACGTGGTGGTGTTCGTGAGCGGCGTGGTGGGCAACGTCACGGTGTGCCTGGTGATTGCGCGCAACTCTCACTTCCAGACACCCACCAACTACTACCTCTTCTCGCTAGCCATCTCGGATCTGCTCATACTCGTCTTCGGTCTGCCCAATGACCTGAAGCTCTACTGGCAACAGTACCCATGGCGACTGGGCGAAGCTCTGTGTCGCTTCAGGGCCTTGGTGGCAGAAGCCACATCGTACGCCTCAGTATTGACCATCGTGGCGTTCACTGCTGAACGTTACGTGGCCATCTATCATCCTCTATTCCTACAGACCACATCGAGCCTGACTCGGGCCGTGCGCATCATCGCCATCATCTGGGTGGTGTCGCTGGTGAGCGCCATACCGTTCGCCATCTACACTCGGGTCAACTTCGTGGACTTCCCTGTTGGCTCGGGTCGCGTGGTGCCCGAGTCAGCTTTCTGTGCCCTACCGATGGACACGACTGCGGTCAGCCTTCCCCTGCTGCAGTGCTCGACGTTCGCCTTCTTCTGCCTTCCCATGACAGTCATAGCAGTGCTGTACCTCAAGATAGGAATGCGGTTAAGGAGCCAGCCAGGACCCGGGCAGCAGGGGAGGCACCAGCGCAGACCAGTGCACAGAATGCTAGTGGCTGTGGTCATAGCGTTCTTCGTCTGCTGGGCTCCGTTCCACACCCAGCGCCTTCTGGTGGTGTACGTCAGCCCCACCCAATGGACTACGGGCCTACGCACTCTCAACGAAGTGCTCTACTACACAGCTGGGTGTCTCTACTACTTCAGTGCCACAATTAACCCGATACTCTACTCGCTGATGTCCGTCAAGTACCGAGAGGCATTTCGCGACGCCCTCTGCACCCTGTCCAAGAATAAGCAGCGGCTCTCAGCTGGAGATTTCGATGCCGGTGCCACCGTAGTTATCGTCGGTGCTGGCCACTCTATATTGGACAACACAAGGCTGAGCACGCTAAAGCCATACTCGATCGTCCAGCGCGTTGACACCGAGGAGGCTAACGTGGACACCGCCGCGGACATAGCGATGCGGGCCCTCATCATCCAGACGGTGCTCCGCGTTAGGCCTAACCAGGAACCGCCACTGACGCCAAGTATGAGCGAAGCTGATGATAAGGAGAAAATTCCCATCAAAGAGATCTCCTCCAGTGACAAGAGCCCCCTGCCTTCGGAGACGGTGGTGTGACTCTCCACCTTGGTGACTCTTGTAGGCATAAACGCCATACCCTGTTC

**RACE-PCR**

**5’ RACE (extended 5’-UTR Cap2b/PVK receptor region, GenBank OP191701)**

The sequence fragment that overlaps with clones #4 and 6# at the 5’ end is highlighted in **bold purple letters**, showing differences only for a few nucleotides, not highlighted.

AGAGCCGGGAAGAGGTTGCAAAACTTTAGCGCCTCCCGGTGTTGAAGGAGGAGCAAAAAAAGAGAAGCGACGACGCGCGACCGCGCCGCTGGGTTTGAACTCGAGCTTGGCACTGACGCTAAGCGCTGAAACCGCGGTGTCGTGACGTTGTCTCGTCTTAACTGCTCGGAGGGACTTCGTCTACGTGAGTTGTTTGTTGGATACCCGCGTGCGAACTGTGGTGGCCTTTGTGTTTGACGTGTCACTCTTTCTCCGATTCTTCGCCCGAAAGGAATTTCGCGGTGTCCCACTTGGGCAGCAGTCAAAACAAATACGTCGGCAAGCCTTGCATCGTCATCATTGACTTGTGGGGTTTTCCCTGTTTCCTGCTGCGCTTCAAGAGCCAAAAAACTCCACGAAAGGCGCCCATGTTTGGTAGATCCATGGCAGAAAACGGTGACCTCGGCTTGACGGCTGATTCCGCGCCTGGAACTCCGCGTCTCTTGTTCTTCGTGACGGAACACAGTTTCTGAACACAGCGAGACCCACCTAAATAAGCGACATCACCAATAAGTGGAAGCAGTAAGATGACTTGAAACTGTGCAAACAGTGCTGGACTTCTTACTCTGATTATCAAGCGTTGAATGCTAAGTACCCAGCGTGGAATGCCTGTGGCGTTGAGTTCGAAGCTTTAGAACAAACGCTTGACAACGTTCTCTAACATAGAAAAACGCGTTGTATTTTCACAGTCTTCACATCAGGTGTACCAACACCTTTGAATGCTCAATGTGGACCACTGGTAGCGTTCGGCCGAACACATAGGCGATAACAAAGTGTTCAATGTTAACCGTAGCAGGTGCGACAGTTAAATTCACACAAAATTACTGAACCTTCTTCAAAGCATCGCCACTGATAGCTCAGGGTCTCTAGTTCACATTTTTGTCCGTGCTTAAGATATAAGGAGCTAGCTGTTCGTTGATTAGTGGAAAAAGGTGTAAACTAAACCTCTGATATCAACTGCTATTTTGAATGCGAAGCGCCGACTATCTGCAGCTTGTCAAATTTGTGGTATATTCTCTGGCTATCTCCCTCCTTATAGTGCCTACTAGCTGCTTATTCGGCTGATAGAGGCAGTCTGGTTTATTCGTTTTATGCAGAAAAACGACTGAGTGGTTTATTAAATTTGTGACCCTTGCGAGAACTTAAATGTGATCTGGGAATTAAAACAAGCGGTGCCTGACACAATGACACGGAAAAGCAATCACACTACCTTAAACTTTTCTCGCATTCCCCGCAAAGAACGTCAAAACCTTTCCACTGGTTGGACCCATTTAGGAACTGCCAGAGTCGTCTGGCTTCTAAATTTTGTTCTTGTACCAAGAACTGCTGCGCGCTCTGTATTGTCGAAAGGACACAAGTTAGTCAATTTAGGAGACGTCAATTACCCGCTTCAAGCTGCATGTGTGTCCTTCCACAGGCGTTTCGAATTGAAAGAACGGAAATGTTTTTCTGTTGCAAACGTCACCGTGTAACCGCTCAAAGCGTTTCGGTCTAACAGGGTGTCACCATAAAGTGCATGGTGGACGGGCAGTTTCAAATACCGGTCATTGTGAACCGAGCATGAACGCTTCGAACTGAGCAGACACCATCACGAAAGGCAACTGGTACAAGGTAGTTGATGTAAGCCTAATAGTGGCACCTCGCTTAGAAACTATCACTTAAGTGAAGGTTTCGCTTGAAGAATCTGTGAGTTATGTGACACTGTCGTCAGGGACGTCAAGCCAAAGAATGTTCTCACCATACTCTGTGATCACCTGTGTATGTCACTAAGAAGAAACTACCAAGAAATAGCTGCGCCTTCGCTGGACT**ATCTGCTGTGCCGACACTGTGAA**AA**GTGTCAGGTTGTGATGCGTAGGCT**T**GGTCACATCGTGA**GT**AC**G**GTGCATCGAGAACACTGAAATAGG**A**CAGATGATTAT**T**CTTG**TA**GTGCTGTGAGTTCCGTGCATC**T**AGGACAGTGCACTGTGATGGAGAACGTAGCGGTGGCAGC**T**GTGAGTTCGTTGGAGGACAACGCCACCATACGGGAGTACTTGGAACTCAGGCTGGGACCCCAGCACATACTGCTGCC**C**ATCGTGATCCCACTCACGGTTCTCTACGTGGTGGTGTTCGTGAGCGGCGTGGTGGGCAACGTCACGGTGTGCCTGGTGATTGCGCGCAACTCTCACTTCCAGACACCCACCAACTACTACCTCTTCTCGCTAGCCATCTCGGATCTGCTCATACTCGTCTTCGGTCTGCCCAACGACCTGAAGCTCTACTGGCAACAGTACCCATGGCGACTGGGCGA**G**GCTCTGTGTCGCTTCAGGGCCTTGGTGGCAGAAGCCAC**T**TCGTACGCCTCAGTATTG**

**Alignments**

**Alignment between *Rhipicephalus microplus* genome [isolate Rmic-2018 Chromosome 3, ASM1333972v1, NC_051167.1] and *R. microplus* periviserokinin receptor RACE 5’-UTR fragment**

**Range 1**: 183031150 to 183033059

**Neuropeptide CAPA receptor**

Score:3024 bits(1637), Expect:0.0,

Identities:1825/1917(95%), Gaps:10/1917(0%), Strand: Plus/Plus

Query 351 GGTTTTCCCTGTTTCCTGCTGCGCTTCAAGAGCCAAAAAACTCCACGAAAGGCGCCCATG 410

|||||||| |||| ||||||| ||||||||||||| |||||||| ||||||| ||||||

Sbjct 183031150 GGTTTTCCTTGTTCCCTGCTGTGCTTCAAGAGCCAGAAAACTCCCCGAAAGGTACCCATG 183031209

Query 411 TTTGGTAGATCCATGGCAGAAAACGGTGACCTCGGCTTGACGGCTGATTCCGCGCCTGGA 470

||||||||||||||||||||||||||||||||||||||||||||||||||||||||||||

Sbjct 183031210 TTTGGTAGATCCATGGCAGAAAACGGTGACCTCGGCTTGACGGCTGATTCCGCGCCTGGA 183031269

Query 471 ACTCCGCGTCTCTTGTTCTTCGTGACGGAACACAGTTTCTGAACACAGCGAGACCCACCT 530

||||| || |||||||||||||||||||||||||||||||||| |||||||||||| ||

Sbjct 183031270 ACTCCACGCCTCTTGTTCTTCGTGACGGAACACAGTTTCTGAATACAGCGAGACCCGTCT 183031329

Query 531 AAATAAGCGACATCACCAATAAGTGGAAGCAGTAAGATGACTTGAAACTGTGCAAACAGT 590

|| ||||||||||||| ||||||||||||||||||||||||||||||||||||||||||

Sbjct 183031330 AAGCAAGCGACATCACCGATAAGTGGAAGCAGTAAGATGACTTGAAACTGTGCAAACAGT 183031389

Query 591 GCTGGACTTCTTACTCTGATTATCAAGCGTTGAATGCTAAGTACCCAGCGTGGAATGCCT 650

|||||||||||||| |||||||| |||||||||| || || |||||||||||||| ||||

Sbjct 183031390 GCTGGACTTCTTACGCTGATTATGAAGCGTTGAACGCCAAATACCCAGCGTGGAACGCCT 183031449

Query 651 GTGGCGTTGAGTTCGAAGCTTTAGAACAAACGCTTGACAACGTTCTCTAACATAGAAAAA 710

||||||||||||| ||||||||||||||||||||||||||||||||| |||||||| |||

Sbjct 183031450 GTGGCGTTGAGTTTGAAGCTTTAGAACAAACGCTTGACAACGTTCTCCAACATAGAGAAA 183031509

Query 711 CGCGTTGTATTTTCAC--AGTCTTCACATCAGGTGTACCAACACCTTTGAATGCTCAATG 768

|||||||||||||||| ||||||||||||||||||||||||||||||||||||||||||

Sbjct 183031510 CGCGTTGTATTTTCACAAAGTCTTCACATCAGGTGTACCAACACCTTTGAATGCTCAATG 183031569

Query 769 TGGACCACTGGTAGCGTTCGGCCGAACACATAGGCGATAACAAAGTGTTCAATGTTAACC 828

||||||||||||||||||||||||||||||||||||||||| ||||||||||||||||||

Sbjct 183031570 TGGACCACTGGTAGCGTTCGGCCGAACACATAGGCGATAACGAAGTGTTCAATGTTAACC 183031629

Query 829 GTAGCAGGTGCGACAGTTAAATTCACACAAAATTACTGAACCTTCTTCAAAGCATCGCCA 888

|||||||||| |||| ||||||||||| ||||||||||||||||||||||||||||||||

Sbjct 183031630 GTAGCAGGTGTGACAATTAAATTCACA-AAAATTACTGAACCTTCTTCAAAGCATCGCCA 183031688

Query 889 CTGATAGCTCAGGGTCTCTAGTTCACATTTTTGTCCGTGCTTAAGATATAAGGAGCTAGC 948

|||||||||||||||||||||||||||||||||||||||||||| ||||||||| |

Sbjct 183031689 CTGATAGCTCAGGGTCTCTAGTTCACATTTTTGTCCGTGCTTAAAGCATAAGGAGC--G- 183031745

Query 949 TGTTCGTTGATTAGTGGAAAAAGGTGTAAACTAAACCTCTGATATCAACTGCTATTTTGA 1008

||||||||||||| |||||||||||||||||||| ||||||||||||||||| ||||||

Sbjct 183031746 -GTTCGTTGATTAGGGGAAAAAGGTGTAAACTAAAGCTCTGATATCAACTGCTGTTTTGA 183031804

Query 1009 ATGCGAAGCGCCGACTATCTGCAGCTTGTCAAATTTGTGGTATATTCTCTGTCTATCTCC 1068

||||||||||||||||||||||||||||||||||||||||||| |||||| ||||||||

Sbjct 183031805 TTGCGAAGCGCCGACTATCTGCAGCTTGTCAAATTTGTGGTATAGTCTCTGTCTATCTCC 183031864

Query 1069 CTCCTTATAGTGCCTACTAGCTGCTTATTCGGCTGATAGAGGCAGTCTGGTTTATTCGTT 1128

|||| |||||||||||||||||||||||||||||||||||||||||||| ||||||||||

Sbjct 183031865 CTCCGTATAGTGCCTACTAGCTGCTTATTCGGCTGATAGAGGCAGTCTGATTTATTCGTT 183031924

Query 1129 TTATGCAGAAAAACGACTGAGTGGTTTATTAAATTTGTGACCCTTGCGAGAACTTAAATG 1188

||||||||||||||||||||||| |||| ||| |||||||||||||||||||||||||||

Sbjct 183031925 TTATGCAGAAAAACGACTGAGTGTTTTAATAATTTTGTGACCCTTGCGAGAACTTAAATG 183031984

Query 1189 TGATCTGGGAATTAAAACAAGC-GGTGCCTGACACAATGACACGGAAAAGCAATCACACT 1247

|||||||||||||||||||| | |||||||||||||||||||||||||||||||| ||||

Sbjct 183031985 TGATCTGGGAATTAAAACAAACTGGTGCCTGACACAATGACACGGAAAAGCAATCGCACT 183032044

Query 1248 ACCTTAAACTTTTCTCGCATTCCCCGCAAAGAACGTCAAAACCTTTCCACTGGTTGACC 1307

||||||||| |||||||||| |||||||||||||||||||||||||||||||||| |||

Sbjct 183032045 ACCTTAAACGTTTCTCGCATGCCCCGCAAAGAACGTCAAAACCTTTCCACTGGTTGACT 183032103

Query 1308 CATTTAGGAACTGCCAGAGTCGTCTGGCTTCTAAATTTTGTTCTTGTACCAAGAACTGCT 1367

||||||||||||||||||||||||||||||||||||||||||||||||||||||||||||

Sbjct 183032104 CATTTAGGAACTGCCAGAGTCGTCTGGCTTCTAAATTTTGTTCTTGTACCAAGAACTGCT 183032163

Query 1368 GCGCGCTCTGTATTGTCGAAAGGACACAAGTTAGTCAATTTAGGAGACGTCAATTACCCG 1427

||||||||||||||||||||||||||||| |||||||||||||||||| |||||||||||

Sbjct 183032164 GCGCGCTCTGTATTGTCGAAAGGACACAAATTAGTCAATTTAGGAGACTTCAATTACCCG 183032223

Query 1428 CTTCAAGCTGCATGTGTGTCCTTCCACAGGCGTTTCGAATTGAAAGAACGGAAATGTTTT 1487

|||||||||||||||||||||||||||||||||||| ||||||||||||||||| | |||

Sbjct 183032224 CTTCAAGCTGCATGTGTGTCCTTCCACAGGCGTTTCAAATTGAAAGAACGGAAAGGCTTT 183032283

Query 1488 TCTGTTGCAAACGTCACCGTGTAACCGCTCAAAGCGTTTCGGTCTAACAGGGTGTCACCA 1547

|||||||||||||||| |||||||||||||||||||||||||||| | ||||||| ||||

Sbjct 183032284 TCTGTTGCAAACGTCATCGTGTAACCGCTCAAAGCGTTTCGGTCTGAAAGGGTGTAACCA 183032343

Query 1548 TAAAGTGCATGGTGGACGGGCAGTTTCAAATACCGGTCATTGTGAACCGAGCATGAACGC 1607

|| |||||| |||||||||||||| || |||||| |||||||||||||||||||| | ||

Sbjct 183032344 TAGAGTGCAGGGTGGACGGGCAGTCTCCAATACCAGTCATTGTGAACCGAGCATGCATGC 183032403

Query 1608 TTCGAACTGAGCAGACACCATCACGAAGGCAACTGGTACAAGGTAGTTGATGTAAGCCT 1667

||||||||||||||||||||||||| |||||||||||||||||||||||||||||||||

Sbjct 183032404 TTCGAACTGAGCAGACACCATCACGAAGGCAACTGGTACAAGGTAGTTGATGTAAGCCT 183032462

Query 1668 AATAGTGGCACCTCGCTTAGAAACTATCACTTAAGTGAAGGTTTCGCTTGAAGAATCTGT 1727

|||||||||||||| | ||||||||| |||||||||||||||||||||||||||||||||

Sbjct 183032463 AATAGTGGCACCTCACCTAGAAACTACCACTTAAGTGAAGGTTTCGCTTGAAGAATCTGT 183032522

Query 1728 GAGTTATGTGACACTGTCGTCAGGGACGTCAAGCCAAAGAATGTTCTCACCATACTCTGT 1787

||||||||||| |||||||||||||||||||||||||||||| |||||||||||||||||

Sbjct 183032523 GAGTTATGTGAAACTGTCGTCAGGGACGTCAAGCCAAAGAATATTCTCACCATACTCTGT 183032582

Query 1788 GATCACCTGTGTATGTCACTAAGAAGAAACTACCAAGAAATAGCTGCGCCTTCGCTGGAC 1847

|||||||||||||||||||||||||||||| |||||||||||||||||||||||| ||||

Sbjct 183032583 GATCACCTGTGTATGTCACTAAGAAGAAACAACCAAGAAATAGCTGCGCCTTCGCAGGAC 183032642

Query 1848 TATCTGCTGTGCCGACACTGTGAAAAGTGTCAGGTTGTGATGCGTAGGCTTGGTCACATC 1907

||||||||||||||||| |||||| |||||||||||||||||||| |||| |||||||||

Sbjct 183032643 TATCTGCTGTGCCGACAGTGTGAAGAGTGTCAGGTTGTGATGCGTTGGCTCGGTCACATC 183032702

Query 1908 GTGAGTACGGTGCATCGAGAACACTGAAATAGGACAGATGATTATTCTTGTAGTGCTGTG 1967

|||| | |||||||||||| |||||||||||||||||||||| ||||||||||||||||

Sbjct 183032703 GTGATCATGGTGCATCGAGAGCACTGAAATAGGACAGATGATTGTTCTTGTAGTGCTGTG 183032762

Query 1968 AGTTCCGTGCATCTAGGACAGTGCACTGTGATGGAGAACGTAGCGGTGGCAGCTGTGAGT 2027

||||||||||||| |||||||||||||||||||||||||||||| |||||||| ||||||

Sbjct 183032763 AGTTCCGTGCATCGAGGACAGTGCACTGTGATGGAGAACGTAGCTGTGGCAGCCGTGAGT 183032822

Query 2028 TCGTTGGAGGACAACGCCACCATACGGGAGTACTTGGAACTCAGGCTGGGACCCCAGCAC 2087

||||||||||||||||||||||||||||||||||||||||||||||||||||||||||||

Sbjct 183032823 TCGTTGGAGGACAACGCCACCATACGGGAGTACTTGGAACTCAGGCTGGGACCCCAGCAC 183032882

Query 2088 ATACTGCTGCCCATCGTGATCCCACTCACGGTTCTCTACGTGGTGGTGTTCGTGAGCGGC 2147

|||||||||||||||||||||||||||||||| |||||||||||||||||||||||||||

Sbjct 183032883 ATACTGCTGCCCATCGTGATCCCACTCACGGTACTCTACGTGGTGGTGTTCGTGAGCGGC 183032942

Query 2148 GTGGTGGGCAACGTCACGGTGTGCCTGGTGATTGCGCGCAACTCTCACTTCCAGACACCC 2207

||||||||||||||||||||||||||||||||||||||||||||||||||||||||||||

Sbjct 183032943 GTGGTGGGCAACGTCACGGTGTGCCTGGTGATTGCGCGCAACTCTCACTTCCAGACACCC 183033002

Query 2208 ACCAACTACTACCTCTTCTCGCTAGCCATCTCGGATCTGCTCATACTCGTCTTCGGT 2264

|||||||||||||||||||||||||||||||||||||||||||||||||||||||||

Sbjct 183033003 ACCAACTACTACCTCTTCTCGCTAGCCATCTCGGATCTGCTCATACTCGTCTTCGGT 183033059

**Range 2**: 182966601 to 182966935

**Neuropeptide CAPA receptor**

Score:459 bits(248), Expect:4e-126,

Identities:317/349(91%), Gaps:17/349(4%), Strand: Plus/Plus

Query 8 GGAAGAGG-TTGCAAAACTTTAGCGCCTCCCGGTGTTGAAGGAGGAGCAAAAAAAGAGAA 66

|||||||| |||||||||||||||||||||||||||| ||||||||||||| |||||

Sbjct 182966601 GGAAGAGGATTGCAAAACTTTAGCGCCTCCCGGTGTTGAAGGAGGAGCAAAAAA-GAGAA 182966659

Query 67 gcgaCGACGCGCGACCGCGCCGCTGGGTTTGAACTCGAGCTTGGCACTGACGCTAAGCGC 126

| ||| || ||| | || |||||||||||||||||||||||||||||||||||

Sbjct 182966660 --G-CGA----CG---GCG-C-CTTGGTTTGAACTCGAGCTTGGCACTGACGCTAAGCGC 182966707

Query 127 TGAAACCGCGGTGTCGTGACGTTGTCTCGTCTTAACTGCTCGGAGG-GACTTCGTCTACG 185

| || |||||||||||||||||||||||||||||||||||||||| |||||||||||||

Sbjct 182966708 TAAACCCGCGGTGTCGTGACGTTGTCTCGTCTTAACTGCTCGGAGCTGACTTCGTCTACG 182966767

Query 186 TGAGTTGTTTGTTGGATACCCGCGTGCGAACTGTGGTGGCCTTTGTGTTTGACGTGTCAC 245

|||||||||||||||||||||||| |||||||||||| |||| |||||||||||||||||

Sbjct 182966768 TGAGTTGTTTGTTGGATACCCGCGCGCGAACTGTGGTTGCCTGTGTGTTTGACGTGTCAC 182966827

Query 246 TCTTTCTCCGATTCTTCGCCC-GAAAGGAATTTCGCGGTGTCCCACTTGGGCAGCAGTCA 304

|||||||||||||| ||| || ||||||||||||| |||||||||||||||||||||||

Sbjct 182966828 TCTTTCTCCGATTCCTCGACCTGAAAGGAATTTCGTGGTGTCCCACTTGGGCAGCAGTCG 182966887

Query 305 AAACAAATACGTCNGCAAGCCTTGCATCGTCAtcattgacttgtggggt 353

||||||||||||| |||||||||||||||||||||||||||||||||||

Sbjct 182966888 AAACAAATACGTC-GCAAGCCTTGCATCGTCATCATTGACTTGTGGGGT 182966935

**Range 3**: 183056171 to 183056284

**Neuropeptide CAPA receptor**

Score:206 bits(111), Expect:5e-50,

Identities:113/114(99%), Gaps:0/114(0%), Strand: Plus/Plus

Query 2262 GGTCTGCCCAACGACCTGAAGCTCTACTGGCAACAGTACCCATGGCGACTGGGCGAGGCT 2321

|| |||||||||||||||||||||||||||||||||||||||||||||||||||||||||

Sbjct 183056171 GGCCTGCCCAACGACCTGAAGCTCTACTGGCAACAGTACCCATGGCGACTGGGCGAGGCT 183056230

Query 2322 CTGTGTCGCTTCAGGGCCTTGGTGGCAGAAGCCACTTCGTACGCCTCAGTATTG 2375

||||||||||||||||||||||||||||||||||||||||||||||||||||||

Sbjct 183056231 CTGTGTCGCTTCAGGGCCTTGGTGGCAGAAGCCACTTCGTACGCCTCAGTATTG 183056284
