# Supplementary material for: Periviscerokinin (Cap2b; CAPA) receptor silencing in females of Rhipicephalus microplus reduces survival, weight and reproductive output
Source: Parasit Vectors. 2022 Oct 6;15:359. doi: 10.1186/s13071-022-05457-7 (PMC9535995; doi:10.1186/s13071-022-05457-7)
Supplement: Supplementary file 2 — Additional file 2: Data S2. Nucleotide sequences of the Rhimi-CAP2bR (KC614697.1) and the extended Rhimi-CAP2bR 5’-UTR sequence. The dsRNAs used for Rhimi-CAP2bR silencing are displayed on these sequences. Further, an EMBO-Clustal Omega 1.2.4 multiple sequence alignment between KC614697.1 sequence and R. microplus periviscerokinin receptor RACE 5’-UTR fragment (R-5’CAP2bR) (Accession number OP191701) is provided. [file 13071_2022_5457_MOESM2_ESM.docx]

Start and stop codons are highlighted in **underlined bold letters**

**dsRNAs locations**

**Orange letters = 680-805**

**Green = 956-1109**

**Blue letters = 1102-1200**

**qRT-PCR amplicon locations**

**Gray = qRT-PCR amplicon**

***R. microplus* extended 5’-UTR Cap2b/PVK receptor region, GenBank is OP191701 (Obtained by RACE-PCR) 2375 bp**

AGAGCCGGGAAGAGGTTGCAAAACTTTAGCGCCTCCCGGTGTTGAAGGAGGAGCAAAAAAAGAGAAGCGACGACGCGCGACCGCGCCGCTGGGTTTGAACTCGAGCTTGGCACTGACGCTAAGCGCTGAAACCGCGGTGTCGTGACGTTGTCTCGTCTTAACTGCTCGGAGGGACTTCGTCTACGTGAGTTGTTTGTTGGATACCCGCGTGCGAACTGTGGTGGCCTTTGTGTTTGACGTGTCACTCTTTCTCCGATTCTTCGCCCGAAAGGAATTTCGCGGTGTCCCACTTGGGCAGCAGTCAAAACAAATACGTCGGCAAGCCTTGCATCGTCATCATTGACTTGTGGGGTTTTCCCTGTTTCCTGCTGCGCTTCAAGAGCCAAAAAACTCCACGAAAGGCGCCCATGTTTGGTAGATCCATGGCAGAAAACGGTGACCTCGGCTTGACGGCTGATTCCGCGCCTGGAACTCCGCGTCTCTTGTTCTTCGTGACGGAACACAGTTTCTGAACACAGCGAGACCCACCTAAATAAGCGACATCACCAATAAGTGGAAGCAGTAAGATGACTTGAAACTGTGCAAACAGTGCTGGACTTCTTACTCTGATTATCAAGCGTTGAATGCTAAGTACCCAGCGTGGAATGCCTGTGGCGTTGAGTTCGAAGCTTTAGAACAAACGCTTGACAACGTTCTCTAACATAGAAAAACGCGTTGTATTTTCACAGTCTTCACATCAGGTGTACCAACACCTTTGAATGCTCAATGTGGACCACTGGTAGCGTTCGGCCGAACACATAGGCGATAACAAAGTGTTCAATGTTAACCGTAGCAGGTGCGACAGTTAAATTCACACAAAATTACTGAACCTTCTTCAAAGCATCGCCACTGATAGCTCAGGGTCTCTAGTTCACATTTTTGTCCGTGCTTAAGATATAAGGAGCTAGCTGTTCGTTGATTAGTGGAAAAAGGTGTAAACTAAACCTCTGATATCAACTGCTATTTTGAATGCGAAGCGCCGACTATCTGCAGCTTGTCAAATTTGTGGTATATTCTCTGGCTATCTCCCTCCTTATAGTGCCTACTAGCTGCTTATTCGGCTGATAGAGGCAGTCTGGTTTATTCGTTTTATGCAGAAAAACGACTGAGTGGTTTATTAAATTTGTGACCCTTGCGAGAACTTAAATGTGATCTGGGAATTAAAACAAGCGGTGCCTGACACAATGACACGGAAAAGCAATCACACTACCTTAAACTTTTCTCGCATTCCCCGCAAAGAACGTCAAAACCTTTCCACTGGTTGGACCCATTTAGGAACTGCCAGAGTCGTCTGGCTTCTAAATTTTGTTCTTGTACCAAGAACTGCTGCGCGCTCTGTATTGTCGAAAGGACACAAGTTAGTCAATTTAGGAGACGTCAATTA**CCCGCTTCAAGCTGCATGTGTGTCCTTCCACAGGCGTTTCGAATTGAAAGAACGGAAATGTTTTTCTGTTGCAAACGTCACCGTGTAACCGCTCAAAGCGTTTCGGTCTAACAGGGTGTCACCATAAAGTGCATGGTGGACGGGCAGTTTCAAATACCGGTCATTGTGAACCGAGCATGAACGCTTCGAACTGAGC**AGACACCATCACGAAAGGCAACTGGTACAAGGTAGTTGATGTAAGCCTAATAGTGGCACCTCGCTTAGAAACTATCACTTAAGTGAAGGTTTCGCTTGAAGAATCTGTGAGTTATGTGACACTGTCGTCAGGGACGTCAAGCCAAAGAATGTTCTCACCATACTCTGTGATCACCTGTGTATGTCACTAAGAAGAAACTACCAAGAAATAGCTGCGCCTTCGCTGGACTATCTGCTGTGCCGACACTGTGAAAAGTGTCAGGTTGTGATGCGTAGGCTTGGTCACATCGTGAGTACGGTGCATCGAGAACACTGAAATAGGACAGATGATTATTCTTGTAGTGCTGTGAGTTCCGTGCATCTAGGACAGTGCACTGTG**ATG**GAGAACGTAGCGGTGGCAGCTGTGAGTTCGTTGGAGGACAACGCCACCATACGGGAGTACTTGGAACTCAGGCTGGGACCCCAGCACATACTGCTGCCCATCGTGATCCCACTCACGGTTCTCTACGTGGTGGTGTTCGTGAGCGGCGTGGTGGGCAACGTCACGGTGTGCCTGGTGATTGCGCGCAACTCTCACTTCCAGACACCCACCAACTACTACCTCTTCTCGCTAGCCATCTCGGATCTGCTCATACTCGTCTTCGGTCTGCCCAACGACCTGAAGCTCTACTGGCAACAGTACCCATGGCGACTGGGCGAGGCTCTGTGTCGCTTCAGGGCCTTGGTGGCAGAAGCCACTTCGTACGCCTCAGTATTG

***Rhimi-CAP_2b_R* (GenBank KC614697.1) 1736 bp**

ACATGGGTGATCTAAGCCTAATAGTGGGACCTCACTTAGCAATCACCACTTAAGTGAAGGTTTCGCTTGAAGAATCTGTGAGTTATGTGAAACTGTCGTCAGGGACGTCAAGCCGAAGAATATCTCACCATACTCTGTGGTCACCTGTGTATGTCACTAAGAAGAAACTACCAAGAAATAGCTGCGCCTTCGCAGGACTATCTGCTG**TGCCGACACTGTGAAGGGTGTCAGGTTGTGATGCGTTGGCTCGGTCACATCGGGATCACGGTGCATGGAGAACACTGAAATAGGACAGATGATTATTCTTGTGGTGCTGTGAGTTCCGTGCATCGAGGACAG**TGCACTGTGATGGAGAACGTAGCTGTGGCAGCCGTGAGTTCGTTGGAGGACAACGCCACCATACGGGAGTACTTGGAACTCAGGCTGGGACCCCAGCACATACTGCTGCCAATCGTGATCCCACTCACGGTACTCTACGTGGTGGTGTTCGTGAGCGGCGTGGTGGGCAACGTCACGGTATGCCTGGTGATTGCGCGCAATTCTCACTTCCAGACACCCACCAACTACTACCTCTTCTCGCTAGCCATCTCGGATCTGCTCATACTCGTCTTCGGCCTGCCCAACGACCTGAAGCTCTACTGGCAACAGTACCCATGGCGACTGGGCGAGGCTCTGTGTCGCTTCAGGGCCTTGGTGGCAGAAGCCACTTCGTACGCCTCAGTATTGACCATCGTGGCGTTCACCGCAGAACGTTACGTGGCCATCTATCACCCTCTCTTCCTACAGACCACATCAAGCCTGACTCGGGCCGTGCGCATCATCGCCATCATCTGGGTGGTGTCGCTGGTGAGCGCCATACCGTTCGCCATCTACACTCGGGTCAACTTCGTTGACTTCCCTGTTGGCTCGGGTCGCGTGGTGCCCGAGTCAGCTTTCTGTGCCCTACCGATGGACACGACTGCGGTCAGCCTTCCCCTGCTGCAGTGCTCGACGTTCGCCTTCTTCTGCCTTCCCATGACAGTCATAGCAGTGCTGTACCTCAAGATAGGAATGCGGTTAAGGAGCCAGCCAGGACCCGGGCAGCAGGGGAGGCACCAGCGCAGACCAGTGCACAGAATGCTGGTGGCGGTGGTCATAGCGTTCTTCGTCTGCTGGGCTCCGTTCCACACCCAGCGCCTTCTGGTGGTGTACGTCAGCCCCACCCAATGGACTACGGGCCTACGCACTCTCAACGAAGTGCTCTACTACACAGCCGGGTGTCTCTACTACTTCAGTGCCACAATTAACCCGATACTCTACTCGCTGATGTCCGTCAAGTACCGAGAGGCATTTCGCGACGCTCTCTGCACCCTGTCCAAGAATAAGCAGCGGCTCTCAGCTGGAGATTTCGATGCCGGTGCCACCGTAGTTATCGTTGGTGCCGGCCACTCTATATTGGACAACACGAGGCTGAGCACGCTAAAGCCATACTCGATCGTCCAGCGCGTTGACACCGATGAGGCAAACGTGGACACGGCCGCGGACATAGCGATGCGGGCCCTCATCATCCAGACGGTGCTCCGCGTTAGGCCTAACCAGGAACCGCCATTGACGCCAAGTATGAGCGAAGCTGACGATAAGGAGAAAATTCCCGTCAAGGAGATCTCCTCCAGTGACAAGAGCCCTCTGCCTTCGGAGACGGTGGTG**TGA**CTCTCCACCTTGGTGACTCTTGTAGGCATAAACGCCATACCCTGTCCAAAAAAAAAAAAAAAAAAAAAAA

**Alignments (EMBO-Clustal Omega1.2.4 multiple sequence alignment)**

**Alignment between KC614697.1 and the new *R. microplus* periviserokinin receptor RACE 5’-UTR fragment (R-5’CAP2bR)**

Numbers on the left of R-5’CAP2bR correspond to the order in the assembled cloned sequence that includes the extended 5’UTR starting at 1.

KC614697.1 ------------------------------------------------------------ 0

*R-5’CAP2bR* AGAGCCGGGAAGAGGTTGCAAAACTTTAGCGCCTCCCGGTGTTGAAGGAGGAGCAAAAAA 60

KC614697.1 ------------------------------------------------------------ 0

*R-5’CAP2bR* AGAGAAGCGACGACGCGCGACCGCGCCGCTGGGTTTGAACTCGAGCTTGGCACTGACGCT 120

KC614697.1 ------------------------------------------------------------ 0

*R-5’CAP2bR* AAGCGCTGAAACCGCGGTGTCGTGACGTTGTCTCGTCTTAACTGCTCGGAGCGGACTTCG 180

KC614697.1 ------------------------------------------------------------ 0

*R-5’CAP2bR* TCTACGTGAGTTGTTTGTTGGATACCCGCGTGCGAACTGTGGTGGCCTTTGTGTTTGACG 240

KC614697.1 ------------------------------------------------------------ 0

*R-5’CAP2bR* TGTCACTCTTTCTCCGATTCTTCGCCCGAAAGGAATTTCGCGGTGTCCCACTTGGGCAGC 300

KC614697.1 ------------------------------------------------------------ 0

*R-5’CAP2bR* AGTCAAAACAAATACGTCGGCAAGCCTTGCATCGTCATCATTGACTTGTGGGGTTTTCCC 360

KC614697.1 ------------------------------------------------------------ 0

*R-5’CAP2bR* TGTTTCCTGCTGCGCTTCAAGAGCCAAAAAACTCCACGAAAGGCGCCCATGTTTGGTAGA 420

KC614697.1 ------------------------------------------------------------ 0

*R-5’CAP2bR* TCCATGGCAGAAAACGGTGACCTCGGCTTGACGGCTGATTCCGCGCCTGGAACTCCGCGT 480

KC614697.1 ------------------------------------------------------------ 0

*R-5’CAP2bR* CTCTTGTTCTTCGTGACGGAACACAGTTTCTGAACACAGCGAGACCCACCTAAATAAGCG 540

KC614697.1 ------------------------------------------------------------ 0

*R-5’CAP2bR* ACATCACCAATAAGTGGAAGCAGTAAGATGACTTGAAACTGTGCAAACAGTGCTGGACTT 600

KC614697.1 ------------------------------------------------------------ 0

*R-5’CAP2bR* CTTACTCTGATTATCAAGCGTTGAATGCTAAGTACCCAGCGTGGAATGCCTGTGGCGTTG 660

KC614697.1 ------------------------------------------------------------ 0

*R-5’CAP2bR* AGTTCGAAGCTTTAGAACAAACGCTTGACAACGTTCTCTAACATAGAAAAACGCGTTGTA 720

KC614697.1 ------------------------------------------------------------ 0

*R-5’CAP2bR* TTTTCACAGTCTTCACATCAGGTGTACCAACACCTTTGAATGCTCAATGTGGACCACTGG 780

KC614697.1 ------------------------------------------------------------ 0

*R-5’CAP2bR* TAGCGTTCGGCCGAACACATAGGCGATAACAAAGTGTTCAATGTTAACCGTAGCAGGTGC 840

KC614697.1 ------------------------------------------------------------ 0

*R-5’CAP2bR* GACAGTTAAATTCACACAAAATTACTGAACCTTCTTCAAAGCATCGCCACTGATAGCTCA 900

KC614697.1 ------------------------------------------------------------ 0

*R-5’CAP2bR* GGGTCTCTAGTTCACATTTTTGTCCGTGCTTAAGATATAAGGAGCTAGCTGTTCGTTGAT 960

KC614697.1 ------------------------------------------------------------ 0

*R-5’CAP2bR* TAGTGGAAAAAGGTGTAAACTAAACCTCTGATATCAACTGCTATTTTGAATGCGAAGCGC 1020

KC614697.1 ------------------------------------------------------------ 0

*R-5’CAP2bR* CGACTATCTGCAGCTTGTCAAATTTGTGGTATATTCTCTGGCTATCTCCCTCCTTATAGT 1080

KC614697.1 ------------------------------------------------------------ 0

*R-5’CAP2bR* GCCTACTAGCTGCTTATTCGGCTGATAGAGGCAGTCTGGTTTATTCGTTTTATGCAGAAA 1140

KC614697.1 ------------------------------------------------------------ 0

*R-5’CAP2bR* AACGACTGAGTGGTTTATTAAATTTGTGACCCTTGCGAGAACTTAAATGTGATCTGGGAA 1200

KC614697.1 ------------------------------------------------------------ 0

*R-5’CAP2bR* TTAAAACAAGCGGTGCCTGACACAATGACACGGAAAAGCAATCACACTACCTTAAACTTT 1260

KC614697.1 ------------------------------------------------------------ 0

*R-5’CAP2bR* TCTCGCATTCCCCGCAAAGAACGTCAAAACCTTTCCACTGGTTGGACCCATTTAGGAACT 1320

KC614697.1 ------------------------------------------------------------ 0

*R-5’CAP2bR* GCCAGAGTCGTCTGGCTTCTAAATTTTGTTCTTGTACCAAGAACTGCTGCGCGCTCTGTA 1380

KC614697.1 ------------------------------------------------------------ 0

*R-5’CAP2bR* TTGTCGAAAGGACACAAGTTAGTCAATTTAGGAGACGTCAATTA**CCCGCTTCAAGCTGCA** 1440

KC614697.1 ------------------------------------------------------------ 0

*R-5’CAP2bR* **TGTGTGTCCTTCCACAGGCGTTTCGAATTGAAAGAACGGAAATGTTTTTCTGTTGCAAAC** 1500

KC614697.1 ------------------------------------------------------------ 0

*R-5’CAP2bR* **GTCACCGTGTAACCGCTCAAAGCGTTTCGGTCTAACAGGGTGTCACCATAAAGTGCATGG** 1560

KC614697.1 ------------------------------------------------------------ 0

*R-5’CAP2bR* **TGGACGGGCAGTTTCAAATACCGGTCATTGTGAACCGAGCATGAACGCTTCGAACTGAGC** 1620

KC614697.1 -----------------------------ACATGGGTGATCTAAGCCTAATAGTGGGACC 31

*R-5’CAP2bR* AGACACCATCACGAAAGGCAACTGGTACAAGGTAGTTGATGTAAGCCTAATAGTGGCACC 1680

* * * **** *************** ***

KC614697.1 TCACTTAGCAATCACCACTTAAGTGAAGGTTTCGCTTGAAGAATCTGTGAGTTATGTGAA 91

*R-5’CAP2bR* TCGCTTAGAAACTATCACTTAAGTGAAGGTTTCGCTTGAAGAATCTGTGAGTTATGTGAC 1740

** ***** ** * ********************************************

KC614697.1 ACTGTCGTCAGGGACGTCAAGCCGAAGAAT-ATCTCACCATACTCTGTGGTCACCTGTGT 150

*R-5’CAP2bR* ACTGTCGTCAGGGACGTCAAGCCAAAGAATGTTCTCACCATACTCTGTGATCACCTGTGT 1800

*********************** ****** ***************** **********

KC614697.1 ATGTCACTAAGAAGAAACTACCAAGAAATAGCTGCGCCTTCGCAGGACTATCTGCTGTGC 210

*R-5’CAP2bR* ATGTCACTAAGAAGAAACTACCAAGAAATAGCTGCGCCTTCGCTGGACTATCTGCT**GTGC** 1860

******************************************* ****************

KC614697.1 CGACACTGTGAAGGGTGTCAGGTTGTGATGCGTTGGCTCGGTCACATCGGGATCACGGTG 270

*R-5’CAP2bR* **CGACACTGTGAAGGGTGTCAGGTTGTGATGCGTAGGCTCGGTCACATCGTGATCACAGTG** 1920

********************************* *************** ****** ***

KC614697.1 CATGGAGAACACTGAAATAGGACAGATGATTATTCTTGTGGTGCTGTGAGTTCCGTGCAT 330

*R-5’CAP2bR* **CATCGAGAACACTGAAATAGGGCAGATGATTATCCTTGCGGTGCTGTGAGTTCCGTGCAT** 1980

*** ***************** *********** **** *********************

KC614697.1 CGAGGACAGTGCACTGTG**ATG**GAGAACGTAGCTGTGGCAGCCGTGAGTTCGTTGGAGGAC 390

*R-5’CAP2bR* **CGAGGACAG**TGCACTGTGATGGAGAACGTAGCGGTGGCAGCCGTGAGTTCGTTGGAGGAC 2040

******************************** ***************************

KC614697.1 AACGCCACCATACGGGAGTACTTGGAACTCAGGCTGGGACCCCAGCACATACTGCTGCCA 450

*R-5’CAP2bR* AACGCCACCATACGGGAGTACTTGGAACTCAGGCTGGGACCCCAGCACATACTGCTGCCA 2100

************************************************************

KC614697.1 ATCGTGATCCCACTCACGGTACTCTACGTGGTGGTGTTCGTGAGCGGCGTGGTGGGCAAC 510

*R-5’CAP2bR* ATCGTGATCCCACTCACGGTTCTCTACGTGGTGGTGTTCGTGAGCGGCGTGGTGGGCAAC 2160

******************** ***************************************

KC614697.1 GTCACGGTATGCCTGGTGATTGCGCGCAATTCTCACTTCCAGACACCCACCAACTACTAC 570

*R-5’CAP2bR* GTCACGGTGTGCCTGGTGATTGCGCGCAACTCTCACTTCCAGACACCCACCAACTACTAC 2220

******** ******************** ******************************

KC614697.1 CTCTTCTCGCTAGCCATCTCGGATCTGCTCATACTCGTCTTCGGCCTGCCCAACGACCTG 630

*R-5’CAP2bR* CTCTTCTCGCTAGCCATCTCGGATCTGCTCATACTCGTCTTCGGTCTGCCCAATGACCTG 2280

******************************************** ******** ******

KC614697.1 AAGCTCTACTGGCAACAGTACCCATGGCGACTGGGCGAGGCTCTGTGTCGCTTCAGGGCC 690

*R-5’CAP2bR* AAGCTCTACTGGCAACAGTACCCATGGCGACTGGGCGAAGCTCTGTGTCGCTTCAGGGCC 2340

************************************** *********************

KC614697.1 TTGGTGGCAGAAGCCACTTCGTACGCCTCAGTATTGACCATCGTGGCGTTCACCGCAGAA 750

*R-5’CAP2bR* TTGGTGGCAGAAGCCACATCGTACGCCTCAGTATTG------------------------ 2400

************************************

KC614697.1 CGTTACGTGGCCATCTATCACCCTCTCTTCCTACAGACCACATCAAGCCTGACTCGGGCC 810

*R-5’CAP2bR* ------------------------------------------------------------ 2460

KC614697.1 GTGCGCATCATCGCCATCATCTGGGTGGTGTCGCTGGTGAGCGCCATACCGTTCGCCATC 870

*R-5’CAP2bR* ------------------------------------------------------------ 2520

KC614697.1 TACACTCGGGTCAACTTCGTTGACTTCCCTGTTGGCTCGGGTCGCGTGGTGCCCGAGTCA 930

*R-5’CAP2bR* ------------------------------------------------------------ 2580

KC614697.1 GCTTTCTGTGCCCTACCGATGGACACGACTGCGGTCAGCCTTCCCCTGCTGCAGTGCTCG 990

*R-5’CAP2bR* ------------------------------------------------------------ 2640

KC614697.1 ACGTTCGCCTTCTTCTGCCTTCCCATGACAGTCATAGCAGTGCTGTACCTCAAGATAGGA 1050

*R-5’CAP2bR* ------------------------------------------------------------ 2700

KC614697.1 ATGCGGTTAAGGAGCCAGCCAGGACCCGGGCAGCAGGGGAGGCACCAGCGCAGACCAGTG 1110

*R-5’CAP2bR* ------------------------------------------------------------ 2760

KC614697.1 CACAGAATGCTGGTGGCGGTGGTCATAGCGTTCTTCGTCTGCTGGGCTCCGTTCCACACC 1170

*R-5’CAP2bR* ------------------------------------------------------------ 2820

KC614697.1 CAGCGCCTTCTGGTGGTGTACGTCAGCCCCACCCAATGGACTACGGGCCTACGCACTCTC 1230

*R-5’CAP2bR* ------------------------------------------------------------ 2880

KC614697.1 AACGAAGTGCTCTACTACACAGCCGGGTGTCTCTACTACTTCAGTGCCACAATTAACCCG 1290

*R-5’CAP2bR* ------------------------------------------------------------ 2940

KC614697.1 ATACTCTACTCGCTGATGTCCGTCAAGTACCGAGAGGCATTTCGCGACGCTCTCTGCACC 1350

*R-5’CAP2bR* ------------------------------------------------------------ 3000

KC614697.1 CTGTCCAAGAATAAGCAGCGGCTCTCAGCTGGAGATTTCGATGCCGGTGCCACCGTAGTT 1410

*R-5’CAP2bR* ------------------------------------------------------------ 3060

KC614697.1 ATCGTTGGTGCCGGCCACTCTATATTGGACAACACGAGGCTGAGCACGCTAAAGCCATAC 1470

*R-5’CAP2bR* ------------------------------------------------------------ 3120

KC614697.1 TCGATCGTCCAGCGCGTTGACACCGATGAGGCAAACGTGGACACGGCCGCGGACATAGCG 1530

*R-5’CAP2bR* ------------------------------------------------------------ 3180

KC614697.1 ATGCGGGCCCTCATCATCCAGACGGTGCTCCGCGTTAGGCCTAACCAGGAACCGCCATTG 1590

*R-5’CAP2bR* ------------------------------------------------------------ 3240

KC614697.1 ACGCCAAGTATGAGCGAAGCTGACGATAAGGAGAAAATTCCCGTCAAGGAGATCTCCTCC 1650

*R-5’CAP2bR* ------------------------------------------------------------ 3300

KC614697.1 AGTGACAAGAGCCCTCTGCCTTCGGAGACGGTGGTG**TGA**CTCTCCACCTTGGTGACTCTT 1710

*R-5’CAP2bR* ------------------------------------------------------------ 3360

KC614697.1 GTAGGCATAAACGCCATACCCTGTCCAAAAAAAAAAAAAAAAAAAAAAA----------- 1759

*R-5’CAP2bR* ------------------------------------------------------------ 3420
