# Supplementary material for: Periviscerokinin (Cap2b; CAPA) receptor silencing in females of Rhipicephalus microplus reduces survival, weight and reproductive output
Source: Parasit Vectors. 2022 Oct 6;15:359. doi: 10.1186/s13071-022-05457-7 (PMC9535995; doi:10.1186/s13071-022-05457-7)
Supplement: Supplementary file 3 — Additional file 3: Data S3. NCBI-BLASTn searches to check for possible off-target effects of the Rhimi-CAP2bR dsRNA sequences. Only two short identical sequences to ds956-1109 were found to be ≤ 15 nt in length, which is not sufficient to cause off-target RNAi effects. [file 13071_2022_5457_MOESM3_ESM.docx]

**BLASTn searches of the *Rhipicephalus microplus* genome for all *Rhimi-CAP_2b_R* dsRNAs tested in the present work.**

Database: NCBI - Genome (ASM1333972v1 reference Annotation Release 100) *Rhipicephalus microplus* ASM1333972v1 [GCF_013339725.1] chromosomes plus unplaced and unlocalized scaffolds (reference assembly in Annotation Release 100). Optimize for: Somewhat similar sequences (blastn).

Query #1: **ds680-805** Query ID: lcl|Query_148887 Length: 196

Sequences producing significant alignments:

Alignments:

1) *Rhimi-CAP_2b_R*

>*Rhipicephalus microplus* isolate Rmic-2018 chromosome 3, ASM1333972v1

Sequence ID: NC_051167.1 Length: 238537928

Range 1: 183032220 to 183032415

Score:291 bits(322), Expect:7e-77,

Identities:182/196(93%), Gaps:0/196(0%), Strand: Plus/Plus

Query 1 CCCGCTTCAAGCTGCATGTGTGTCCTTCCACAGGCGTTTCGAATTGAAAGAACGGAAATG 60

|||||||||||||||||||||||||||||||||||||||| ||||||||||||||||| |

Sbjct 183032220 CCCGCTTCAAGCTGCATGTGTGTCCTTCCACAGGCGTTTCAAATTGAAAGAACGGAAAGG 183032279

Query 61 TTTTTCTGTTGCAAACGTCACCGTGTAACCGCTCAAAGCGTTTCGGTCTAACAGGGTGTC 120

||||||||||||||||||| |||||||||||||||||||||||||||| | |||||||

Sbjct 183032280 CTTTTCTGTTGCAAACGTCATCGTGTAACCGCTCAAAGCGTTTCGGTCTGAAAGGGTGTA 183032339

Query 121 ACCATAAAGTGCATGGTGGACGGGCAGTTTCAAATACCGGTCATTGTGAACCGAGCATGA 180

|||||| |||||| |||||||||||||| || |||||| ||||||||||||||||||||

Sbjct 183032340 ACCATAGAGTGCAGGGTGGACGGGCAGTCTCCAATACCAGTCATTGTGAACCGAGCATGC 183032399

Query 181 ACGCTTCGAACTGAGC 196

| ||||||||||||||

Sbjct 183032400 ATGCTTCGAACTGAGC 183032415

2) No Off-target hits.

Query #2: **ds956-1109** Query ID: lcl|Query_148888 Length: 173

Sequences producing significant alignments:

Alignments:

1) *Rhimi-CAP_2b_R*

>*Rhipicephalus microplus* isolate Rmic-2018 chromosome 3, ASM1333972v1

Sequence ID: NC_051167.1 Length: 238537928

Range 1: 183032497 to 183032667

Score:287 bits(317), Expect:3e-75,

Identities:166/171(97%), Gaps:0/171(0%), Strand: Plus/Plus

Query 1 GTGAAGGTTTCGCTTGAAGAATCTGTGAGTTATGTGACACTGTCGTCAGGGACGTCAAGC 60

||||||||||||||||||||||||||||||||||||| ||||||||||||||||||||||

Sbjct 183032497 GTGAAGGTTTCGCTTGAAGAATCTGTGAGTTATGTGAAACTGTCGTCAGGGACGTCAAGC 183032556

Query 61 CAAAGAATGTTCTCACCATACTCTGTGATCACCTGTGTATGTCACTAAGAAGAAACTACC 120

|||||||| ||||||||||||||||||||||||||||||||||||||||||||||| |||

Sbjct 183032557 CAAAGAATATTCTCACCATACTCTGTGATCACCTGTGTATGTCACTAAGAAGAAACAACC 183032616

Query 121 AAGAAATAGCTGCGCCTTCGCTGGACTATCTGCTGTGCCGACACTGTGAAG 171

||||||||||||||||||||| ||||||||||||||||||||| |||||||

Sbjct 183032617 AAGAAATAGCTGCGCCTTCGCAGGACTATCTGCTGTGCCGACAGTGTGAAG 183032667

2) Other identical short sequence

>*Rhipicephalus microplus* isolate Rmic-2018 chromosome 5, ASM1333972v1

Sequence ID: NC_051169.1 Length: 206569738

Range 1: 19890016 to 19890048

Score:43.7 bits(47), Expect:0.048,

Identities:30/33(91%), Gaps:1/33(3%), Strand: Plus/Minus

Query 44 CGTCAGGG-ACGTCAAGCCAAAGAATGTTCTCA 75

|||||||| ||||||||||||||| |||||||

Sbjct 19890048 CGTCAGGGCACGTCAAGCCAAAGACAGTTCTCA 19890016

3) Other identical short sequence

>*Rhipicephalus microplus* isolate Rmic-2018 chromosome 7, ASM1333972v1

Sequence ID: NC_051171.1 Length: 175432524

Range 1: 102567454 to 102567486

Score:43.7 bits(47), Expect:0.048,

Identities:30/33(91%), Gaps:1/33(3%), Strand: Plus/Minus

Query 44 CGTCAGGG-ACGTCAAGCCAAAGAATGTTCTCA 75

|||||||| ||||||||||||||| |||||||

Sbjct 102567486 CGTCAGGGCACGTCAAGCCAAAGACAGTTCTCA 102567454

Query #3: **ds1102-1200** Query ID: lcl|Query_148889 Length: 133

Alignments:

1) *Rhimi-CAP_2b_R*

>*Rhipicephalus microplus* isolate Rmic-2018 chromosome 3, ASM1333972v1

Sequence ID: NC_051167.1 Length: 238537928

Range 1: 183032651 to 183032783

Score:191 bits(211), Expect:1e-46,

Identities:122/133(92%), Gaps:0/133(0%), Strand: Plus/Plus

Query 1 GTGCCGACACTGTGAAGGGTGTCAGGTTGTGATGCGTAGGCTCGGTCACATCGTGATCAC 60

||||||||| ||||||| ||||||||||||||||||| |||||||||||||||||||||

Sbjct 183032651 GTGCCGACAGTGTGAAGAGTGTCAGGTTGTGATGCGTTGGCTCGGTCACATCGTGATCAT 183032710

Query 61 AGTGCATCGAGAACACTGAAATAGGGCAGATGATTATCCTTGCGGTGCTGTGAGTTCCGT 120

||||||||||| |||||||||||| ||||||||| | |||| ||||||||||||||||

Sbjct 183032711 GGTGCATCGAGAGCACTGAAATAGGACAGATGATTGTTCTTGTAGTGCTGTGAGTTCCGT 183032770

Query 121 GCATCGAGGACAG 133

|||||||||||||

Sbjct 183032771 GCATCGAGGACAG 183032783

2) No Off-target hits.
